# Population Health Metrics Research Consortium gold standard verbal autopsy validation study: design, implementation, and development of analysis datasets

**DOI:** 10.1186/1478-7954-9-27

**Published:** 2011-08-04

**Authors:** Christopher JL Murray, Alan D Lopez, Robert Black, Ramesh Ahuja, Said Mohd Ali, Abdullah Baqui, Lalit Dandona, Emily Dantzer, Vinita Das, Usha Dhingra, Arup Dutta, Wafaie Fawzi, Abraham D Flaxman, Sara Gómez, Bernardo Hernández, Rohina Joshi, Henry Kalter, Aarti Kumar, Vishwajeet Kumar, Rafael Lozano, Marilla Lucero, Saurabh Mehta, Bruce Neal, Summer Lockett Ohno, Rajendra Prasad, Devarsetty Praveen, Zul Premji, Dolores Ramírez-Villalobos, Hazel Remolador, Ian Riley, Minerva Romero, Mwanaidi Said, Diozele Sanvictores, Sunil Sazawal, Veronica Tallo

**Affiliations:** 1 Institute for Health Metrics and Evaluation, University of Washington, 2301 Fifth Ave, Suite 600, Seattle, WA 98121, USA; 2 University of Queensland, School of Population Health, Brisbane, Australia; 3 Johns Hopkins University, Bloomberg School of Public Health, Baltimore, MD, USA; 4 Community Empowerment Lab, Shivgarh, India, and The INCLEN Trust International, New Delhi, India; 5 Public Health Laboratory-IdC, Pemba, Tanzania; 6 Public Health Foundation of India, New Delhi, India; 7 Brigham and Women's Hospital, Boston, MA, USA; 8 CSM Medical University, Lucknow, India; 9 Harvard University, School of Public Health, Boston, MA, USA; 10 National Institute of Public Health, Cuernavaca, Mexico; 11 The George Institute for Global Health, Camperdown, Australia; 12 Research Institute for Tropical Medicine, Manila, Philippines; 13 Cornell University, Division of Nutritional Sciences, Ithaca, NY, USA; 14 The George Institute for Global Health, India, Hyderabad, India; 15 Muhimbili University of Health and Allied Sciences, Dar es Salaam, Tanzania

**Keywords:** Verbal autopsy, VA, validation, Philippines, Tanzania, India, Mexico, gold standard, cause of death

## Abstract

**Background:**

Verbal autopsy methods are critically important for evaluating the leading causes of death in populations without adequate vital registration systems. With a myriad of analytical and data collection approaches, it is essential to create a high quality validation dataset from different populations to evaluate comparative method performance and make recommendations for future verbal autopsy implementation. This study was undertaken to compile a set of strictly defined gold standard deaths for which verbal autopsies were collected to validate the accuracy of different methods of verbal autopsy cause of death assignment.

**Methods:**

Data collection was implemented in six sites in four countries: Andhra Pradesh, India; Bohol, Philippines; Dar es Salaam, Tanzania; Mexico City, Mexico; Pemba Island, Tanzania; and Uttar Pradesh, India. The Population Health Metrics Research Consortium (PHMRC) developed stringent diagnostic criteria including laboratory, pathology, and medical imaging findings to identify gold standard deaths in health facilities as well as an enhanced verbal autopsy instrument based on World Health Organization (WHO) standards. A cause list was constructed based on the WHO Global Burden of Disease estimates of the leading causes of death, potential to identify unique signs and symptoms, and the likely existence of sufficient medical technology to ascertain gold standard cases. Blinded verbal autopsies were collected on all gold standard deaths.

**Results:**

Over 12,000 verbal autopsies on deaths with gold standard diagnoses were collected (7,836 adults, 2,075 children, 1,629 neonates, and 1,002 stillbirths). Difficulties in finding sufficient cases to meet gold standard criteria as well as problems with misclassification for certain causes meant that the target list of causes for analysis was reduced to 34 for adults, 21 for children, and 10 for neonates, excluding stillbirths. To ensure strict independence for the validation of methods and assessment of comparative performance, 500 test-train datasets were created from the universe of cases, covering a range of cause-specific compositions.

**Conclusions:**

This unique, robust validation dataset will allow scholars to evaluate the performance of different verbal autopsy analytic methods as well as instrument design. This dataset can be used to inform the implementation of verbal autopsies to more reliably ascertain cause of death in national health information systems.

## Background

Verbal autopsy (VA) is a critically important tool to measure causes of death in populations without complete medical certification of causes of death. A variety of methods have been proposed for VA cause assignment [1,2], ranging from physician-certified verbal autopsy (PCVA) [3,4] to data-derived algorithms [5-7], various applications of Bayes' theorem [8-13], and direct statistical estimation of cause fractions [14]. New methods to analyze VAs and attribute causes of death to them are now being developed [15-19], and it is likely that there will continue to be new methods and refinements. Given both the increasing demand for good cause of death information for the world's poorest populations and the expanding array of VA approaches, it is essential to be able to assess the performance of these options in a scientific and comparable manner.

Several validation studies of VA cause assignment methods have been published [2,3,12,20-31]. Results of validation studies to date, however, have been challenged on several grounds [32-34]. First, previously published validation studies compare the cause of death for individuals derived from verbal autopsy to the cause of death recorded in hospital records or that derived from independent review of hospital medical records. The quality of record keeping and the laboratory, medical imaging, and pathological services available in many developing country hospitals can be extremely poor. This is especially true in resource-poor remote areas where validation studies have been undertaken. As a result, many of these validation studies are actually comparisons of two imperfect cause of death assignment approaches: low-quality hospital-assigned cause of death and the verbal autopsy. In the language of psychometrics, most studies provide information on convergent validity rather than a comparison to a true gold standard known as criterion validity [35]. Second, many studies start with a community sample and then trace back as many deaths to hospital records as possible. The resulting studies often yield small numbers for many causes, so that published results only cover the convergent validity of VA with hospital-assigned (or derived) causes of death for a limited number of causes of death. For many important causes of death such as liver cirrhosis, chronic obstructive pulmonary disease (COPD), or specific sites of cancer, there is essentially no published information on performance of VA. Third, validation studies often do not provide details on the exact items in the VA instrument, the training of interviewers, the training of physicians for PCVA, the coding of death certificates completed by physicians for PCVA, or the protocol used to extract a cause of death from the hospital records.

The Population Health Metrics Research Consortium (PHMRC) gold standard verbal autopsy validation study was initiated in 2005 to address these research limitations and to ensure that comparative assessments of VA performance were based on clinically reliable diagnoses. We designed the study as a multisite collaboration that aims to address some of the key limitations of previous validation studies and stimulate the development of new methods or refinements of existing methods. The primary goal was to collect a dataset that would help provide more definitive answers as to which VA approaches are more valid and to capture data in a standardized way. In this paper, we describe the design of the study, the criteria used to establish a gold standard (GS) cause of death, the implementation of fieldwork, and the creation of standardized datasets for developing and testing new methods.

## Methods

### Data collection sites

Gold standard VA data collection was implemented in six sites in four countries: Andhra Pradesh, India; Bohol, Philippines; Dar es Salaam, Tanzania; Mexico City, Mexico; Pemba Island, Tanzania; and Uttar Pradesh, India. Table 1 shows the age and sex distribution for the decedents represented in this study, as well as the national life expectancy.

**Table 1:** The age and sex distribution of the decedents represented in the verbal autopsy sample and the national life expectancy for the country according to the 2010 United Nations numbers

Site	National life expectancy	Decedents sampled				
		
		% Male	% Female	% Under age 5	% Ages 5 - 59	% Ages 60+
**Andhra Pradesh, India**	64.2	59	41	28	55	17

**Bohol, Philippines**	67.8	56	44	31	38	31

**Dar es Salaam, Tanzania**	55.4	48	52	44	41	15

**Federal District and Morelos, Mexico**	76.2	53	46	21	46	34

**Pemba Island, Tanzania**	55.4	52	48	60	31	10

**Uttar Pradesh, India**	64.2	58	42	24	58	18

Research at the Andhra Pradesh, India, site was implemented and coordinated through the George Institute for Global Health, India, and was centered in the main capital city, Hyderabad, as well as the neighboring areas of Ranga Reddy, Medak, and Nalgonda. Hyderabad is 100% urban with a population of roughly 3,830,000 inhabitants. The neighboring area Ranga Reddy has a similar population size (3,575,000) and is roughly half urban and half rural. The Medak and Nalgonda areas are similar to each other, both roughly 14% urban, comprised of 3,248,000 people in Nalgonda and 2,670,000 in Medak.

The Bohol Island site was led by the Research Institute for Tropical Medicine in Manila. Bohol is a tropical island province located in the Central Visayas of the Philippines, with 46 municipalities and Tagbilaran City. Verbal autopsies were collected over the entire island, as well as a small proportion from Manila. According to the 2007 census, 1,230,000 people live in Bohol. Manila is urban, while Bohol is divided into roughly 46% urban and 54% rural.

The research site in Dar es Salaam, Tanzania, was managed by collaborators at the Muhimbili University of Health and Allied Sciences. Verbal autopsies were collected from all over the city of Dar es Salaam, which has a population of roughly 2,487,000 people according to the 2002 census, with 94% of people living in urban areas and 6% living in rural areas.

The Mexican study was coordinated by the National Institute of Public Health in the Federal District and the state of Morelos. According to the 2010 Census, 8.85 million inhabitants live in the Federal District and 1.8 million live in Morelos. Sixteen percent of the population of the state lives in rural areas [36].

Pemba Island, Tanzania, is the smaller of the two islands of the Zanzibar archipelago. The research there was coordinated through the Public Health Laboratory Ivo de Carneri as part of a collaboration between the Ministry of Health and Social Welfare and Johns Hopkins University. Verbal autopsies were collected from all areas of the island. This island has a population of roughly 400,000 inhabitants. The island is 99% rural and 1% semi-urban.

Finally, the Uttar Pradesh site in India was led by collaborators at the CSM Medical University (CSMMU, formerly, King George Medical College) in Lucknow. Verbal autopsies were collected from a wide range of districts in the state of Uttar Pradesh: Ambedkar Nagar, Bahraich, Barabanki, Basti, Faizabad, Gonda, Hardoi, Lakhimpur, Lucknow, Rae Bareli, Sitapur, Sultanpur, and Unnao. Table 2 shows the population and urban percentage for each of these districts.

**Table 2:** The population size in thousands and percent of population that is urban for the Uttar Pradesh, India field sites, according to the 2001 Census of India

	Population Size	% Urban
Ambedkar Nagar	2,026	9

Bahraich	2,381	10

Barabanki	2,673	9

Basti	2,084	6

Faizabad	2,088	13

Gonda	2,765	7

Hardoi	3,398	12

Lakhimpur	889	7

Lucknow	3,647	64

Rae Bareli	2,872	10

Sitapur	3,619	12

Sultanpur	3,214	4

Unnao	2,700	15

### Instrument

The instrument development was based on the WHO standardized verbal autopsy instrument [37], which in turn was based in part on the work of Chandramohan et al. (1994) for adult deaths and of Anker et al. (1999) for neonatal and child deaths [38,39]. Separate questions were developed for neonatal deaths and stillbirths, children 1 month to 11 years, and adults 12 years and older. Experience gained from VA studies in Andhra Pradesh and China where the WHO instrument, or slight variants of it, had been applied was also considered [40,41]. A committee drawn from the principal and associate investigators considered modifications based on published and unpublished experiences with the WHO instrument, including fieldwork conducted as part of a large VA study in Thailand. The final instrument was translated into the respective local languages, and then back-translated to English by a different translator to ensure accuracy.

The PHMRC instrument is comprised of a general information module, an adult module, and a child and neonatal module. Skip patterns were integrated into the general information module to collect the age of the deceased and then direct interviewers to the correct module to administer. In administering the WHO instrument, the interviewer must first determine the age of the deceased and select the correct instrument to deliver, which results in the potential for more interviewer error and a less fluid interview. The general information module, which is administered in all verbal autopsies, collects items such as education of the decedent, household characteristics, and a household roster. The adult module collects a history of chronic conditions, symptoms of the deceased, women's health questions if the decedent is female, alcohol and tobacco use, and injury information; it also transcribes any available medical record and death certificate information. The child and neonatal module first asks background questions on information such as whether the mother is still alive, where the deceased was born, the size of the decedent at birth, and the delivery date. The questionnaire then ascertains whether the decedent was a stillbirth and, if so, collects symptom questions, such as signs of injury. If not, the questionnaire collects more general information such as the age of the baby or child when they became ill and the age at death. If the decedent is under 28 days (inclusive of stillbirths), a maternal history is collected. In addition, if the decedent is under 28 days and was born live, a full set of neonatal symptom questions are collected. If the decedent is between 28 days to 11 years, infant and child symptom questions are asked. All available health records and death certificates are transcribed for both neonatal and child deaths. Finally, for all ages, the open narrative section was moved to the end of the interview, after the structured questions. This was done to ensure that in future work, we could remove the open-ended items without concern that the results collected in this study were a function of the open-ended items coming prior to structured content.

In addition to the structural changes, there are important differences between the PHMRC instrument and the WHO instrument. First, the WHO adult module is administered on ages 15 and above, while the PHMRC adult module begins at age 12. This expansion of the ages included in the adult module ensures that conditions clinically present, such as maternal mortality in 12 to 14 year olds, are captured through this instrument. Second, a substantial portion of the questions were reworded to ensure clarity. Medical terminology was converted to easily understandable descriptions to target a lay population. For example, "Did s/he have abdominal distension?" was reworded to "Did [NAME] have a more than usual protruding belly?" Information was also added for precision, or removed to ensure only the most diagnostically relevant information was collected. Similarly, we added or dropped entire questions to capture the most essential information, while reducing the duration of the interview as much as possible. One common question type dropped from the instrument was the duration of certain symptoms. For example, the PHMRC instrument asks whether adults had developed a lump in the neck, armpit, breast, or groin but dropped the follow up question "For how long did s/he have the lumps?" as the presence of the symptom alone was the most important information. Another common question type dropped from the WHO instrument was about treatment that had been received by the decedent, as they were less important in informing the cause of death. Finally, the PHMRC instrument did not include questions about chronic conditions in children, such as cancer, tuberculosis, and diabetes. Additional file 1 illustrates the content questions, such as symptoms experienced by the decedent that were added or dropped when converted from the WHO instrument to the PHMRC instrument. The small wording changes are not included in this additional file, though the full PHMRC instrument is included in Additional file 2 (general module), Additional file 3 (adults), and Additional file 4 (children and neonates) for reference.

### Cause list

A key challenge for the study was to identify the cause list for each of the three age groups for which we would seek to collect a sample of gold standard deaths. Our selection of the target cause list was based on consideration of the WHO estimates of the leading causes of death in the developing world in each age group, those causes for which verbal autopsy might be able to function adequately because unique signs and symptoms could potentially be collected in an interview, and the potential to find, in the six sites, deaths with sufficient laboratory, medical imaging, and pathological detail in order that a gold standard cause of death assignment could be made. The cause lists were also designed so that they were mutually exclusive and collectively exhaustive. The target cause list for adults, children, and neonates included 53, 27, and 13 GS causes, respectively, plus stillbirths (for a complete list of causes, see Additional file 5). These cause lists are much longer than for any previously undertaken VA validation study. In fact, nearly all previous VA validation studies have started with a community or convenience sample of deaths and then ascertained cause in hospital records rather than seeking to collect data on a list of causes by design.

### Gold standard criteria

A critical component of the study was the development, for each cause, of clear criteria that had to be fulfilled for a death to be assigned as a GS cause of death. Depending on the cause of death, these criteria included clinical endpoints, laboratory findings, medical imaging, and pathology. Additional file 6 (adults) and Additional file 7 (children and neonates) provide the gold standard criteria for each cause. These gold standard criteria were developed by a committee of physicians involved in the study and underwent multiple cycles of group review.

Preliminary review of hospital records in the sites indicated it would be very difficult to identify any deaths for some causes that would meet the strict gold standard criteria. In order to ensure that as many potentially eligible deaths in each site as possible were collected for the study, a less strict but nevertheless detailed level 2 set of criteria were also developed (see Additional files 6 and 7). In some cases, these level 2 criteria were further disaggregated into level 2A and level 2B. By way of example, the criteria for determining a death as being due to adult breast cancer, adult acute myocardial infarction, child pneumonia, and neonatal birth asphyxia are shown in Table 3.

**Table 3:** Examples of gold standard criteria for adult breast cancer, adult acute myocardial infarction, child pneumonia, and neonatal birth asphyxia

Adult breast cancer	
Level 1	One of the following:
	• Operative specimen with histological confirmation
	• Biopsy/fine needle aspiration cytology

Level 2A	Both of the following:
	• Mammography diagnosis
	• Imaging evidence of metastases in bone, lung, etc. based on CT scan/MRI/X-rays

Level 2B	Patient under treatment from a recognized cancer hospital or cancer unit for breast cancer in cases where the basis for the initial diagnosis is no longer available.

**Adult acute myocardial infarction**	

Level 1	Evidence of acute MI within three months preceding death based upon one or more of the following:
	• Cardiac perfusion scan
	• ECG changes
	• Documented history of CABG or PTCA or stenting
	• Coronary angiography
	• Enzyme changes (any troponin elevation or CK-MB isoenzyme elevation >2 times the upper limit of normal) in the context of myocardial ischemia

Level 2A	Clinical evidence of the following:
	• Sudden death within six hours of the onset of characteristic shock and chest pain when the case has been witnessed by a physician

**Child pneumonia**	

Level 1	Chest X-ray showing primary end-point consolidation, pleural effusion or other consolidation/infiltration, plus two or more of the following:
	• Respiratory rate >70/minute
	• Severe lower chest indrawing
	• Abnormal breath sounds (i.e., grunting, decreased breath sounds, crepitations)
	• Rectal temperature >38°C or <36°C
	• Oral or axillary temperature >37.5°C or <35.5°C

**Neonatal birth asphyxia**	

Level 1	Each of the following:
	• Failure both to breathe spontaneously and to cry at birth
	• No major congenital abnormality
	• Not a stillbirth (one or more signs of life at birth like pulse or movement)
	Plus one of the following in the 24 hours after birth:
	• Not feeding
	• Hypotonia
	• Seizures
	• Needed and failed resuscitation at birth

Level 1 is the most stringent criteria, while level 2A or 2B were also collected for some causes.

By recording the level of diagnosis for each death, we are able to test whether the assessment of performance for any method is affected by the level of cause of death assignment according to our criteria.

### Data collection

#### Identification of gold standard deaths

As described above, a stringent set of diagnostic criteria for each cause of death was developed by a team of study physicians before fieldwork began. Each site then enrolled local health facilities at which medical records would be reviewed. Consortium members led a two-day training at each of the sites to train the reviewers in the gold standard definitions, the protocols for identifying cases meeting these criteria, and the procedure for extracting the pertinent medical information. Each reviewer was provided a pocket guide detailing the necessary criteria for each gold standard cause of death. The medical information from qualifying records was extracted using a standard medical data extraction form (MDEF, see Additional file 8), which the study team developed. Once eligible records were extracted, a local physician reviewed the medical information and determined the gold standard level of the particular case according to the diagnostic criteria outlined for each level for each cause. The following information details the specific protocol followed by each research site.

In Andhra Pradesh, four hospitals were recruited for the study. Three are government hospitals - Gandhi Hospital, Osmania General Hospital, and Chest Hospital - and one is a private hospital, CARE Foundation. There was 24-hour surveillance at the hospitals and all patients were enrolled with their addresses. Study supervisors collected information on all deceased patients from all wards, and clinicians involved in the study then reviewed the case sheets to select those that conformed to the gold standard criteria (levels 1, 2A, and 2B). The medical information from all qualifying cases selected by the clinicians was extracted and sent to the George Institute Hyderabad office for enrollment in the verbal autopsy study.

In Bohol, the majority of deaths were reviewed at the Bohol Regional Hospital. This facility is the referral hospital for Bohol Province with the highest available standards of clinical investigation and hence diagnosis. Three nurses monitored all deaths in the hospital. They ensured that all reports of investigations (imaging and laboratory) were located and attached to the charts. In addition, to augment the number of deaths collected, 467 deaths were recruited from two hospitals in Manila: the Veterans Memorial Medical Center and the Rizal Medical Center. In all locations, the nurses summarized the case notes, including reports of investigations, onto the medical data extraction forms. MDEFs were first reviewed by two study physicians who assigned cause of death and decided by diagnosis and GS level which VAs should not be collected. Deaths were reviewed as soon as possible after the death.

At the Dar es Salaam site, five health facilities were used as recruitment sites. These were Mwananyamala Hospital, Temeke Hospital, Muhimbili National Hospital, Ocean Road Cancer Institute, and Hindu Mandal Hospital. Mwananyamala and Temeke are both district hospitals, each of which records roughly 1,500 deaths per year. Ocean Road Cancer Institute is the only cancer treatment facility in Tanzania and was an important source for causes such as cervical cancer, esophageal cancer, breast cancer, leukemia, prostate cancer, and lymphomas. Muhimbili National Hospital is a referral and teaching hospital with a higher mortality rate than the other enrolled facilities. Hindu Mandal Hospital is a private hospital in the heart of Dar es Salaam. It has a well-established HIV/AIDS clinic and commonly receives noncommunicable disease cases. At each location, a nurse affiliated with the study reviewed medical records to identify qualifying cases. The cases identified by the nurses were reviewed by physicians, who filled out the MDEFs with the gold standard levels for the cases that were eligible for enrollment. The nurses spoke with family members of the deceased if present at the hospital to enroll them in the study, collect their consent, and obtain mapping information and directions for a verbal autopsy interview.

In Mexico, after obtaining authorization to work in each medical unit, a group of six trained physicians reviewed the medical records of cases (and when available the reports from autopsies) that could be included in the study, filled an extraction form for each case, and classified them as levels 1, 2, or 3 according to the gold standard criteria proposed by the PHMRC. Only cases classified as levels 1 and 2 were considered eligible for the study. The original design considered the inclusion of only one to three large hospitals in Mexico City, but due to the difficulty of completing the quota of gold standard cases, hospitals from the health service network of the Federal District government and from the Ministry of Health of the state of Morelos were included. The data were collected from 36 public hospitals: 33 from the Federal District and three from Morelos.

In Pemba, there are four major government hospitals on the island, though most facilities do not have a certified medical doctor present and are managed by medical assistants and nurses. Surveillance systems were put in place in all four hospitals to identify deaths and to classify them into GS categories. The hospital supervisor recorded complete identification information upon admission of each patient, and the attending physician medical assistant confirmed the admission diagnosis. Hospital supervisors ensured that the signs and symptoms experienced by the patient were recorded and that a mortality form with the cause(s) of death was filled out by the attending physician in the event of a death. All forms were sent back to the field headquarters for data entry. A computer algorithm was run to identify cases meeting GS criteria, and all GS cases were recorded in a database. A computer listing was prepared with identifier information to schedule the VA interviews.

In Uttar Pradesh, the gold standard deaths were enrolled at CSMMU, Lucknow, which is a tertiary care government facility with patient inflow from all over Uttar Pradesh and bordering states, including districts in the neighboring country of Nepal. The catchment area spreads over a radius of more than 500 km, of which about 85% cases come from 13 districts surrounding Lucknow. There was 24-hour surveillance at facilities and all patients were enrolled with an address. When a death occurred, the project medical officer reviewed the patient case sheet in consultation with the resident doctor in order to assess the GS levels against standard criteria.

#### VA interview

Once enrolled, the VA interviewers at each site attended a training session led by consortium members using standardized materials and an interviewer's manual. The training manuals provided information on the study background, the roles and responsibilities of the VA interviewer, background on how VA cases were selected, instructions for administering the questionnaire, and information on every question in the instrument. The manual provided guidance on how to handle an array of questions or concerns, tips for building rapport with the respondents, and probing as needed to collect reliable information.

Following the training, VA assignments were given to interviewers blinded to the medical information or cause of death of the decedent along with directions or map queues to the households. In some sites the families were contacted in advance to schedule an appointment, though this decision was left to the sites' discretion. All interviews were collected after a culturally appropriate grieving period had passed. The minimum grievance period was six days in Bohol and the maximum was six months in Mexico (as required by the ethics boards at the hospitals). The maximum amount of time post-death that an interview was collected was eight months in the Mexico site.

The rate of interview refusals varied by site from 1.8% to 9.5%. For those that consented to a verbal autopsy, the instrument was administered on paper in the field, and returned to the field headquarters for double data entry. Interviews lasted an average of 45 minutes across all of the sites.

#### Quality control of fieldwork and data entry

To ensure the highest quality data was collected, quality control checks were performed both at the individual site level, as well as at the Institute for Health Metrics and Evaluation (IHME), where all data were transmitted through a secured password-protected site for analysis.

In all sites, supervisors were trained in the protocols for monitoring quality control at the site level. Supervisors were instructed to observe VA interviewers in the field during the early stage of data collection to ensure they were conducted properly and to provide guidance. Supervisors additionally checked every VA form collected throughout the study to ensure that it was filled out consistently and correctly. If issues were identified by the supervisor, a reinterview was conducted as needed. The field interviewers had periodic meetings with their supervisors to discuss performance, progress, and challenges. Supervisors at most sites additionally reinterviewed a portion of the verbal autopsies to spot check the quality of the information collected.

At IHME, we systematically evaluated all datasets electronically for numerous types of quality issues by a comprehensive set of codes. First, we reviewed the dataset for missing values and for incorrect skip patterns that result in specific questions having been filled in or left blank erroneously. The dataset was also evaluated to determine if any of the observed values fell outside of expected ranges. For example, if the response for a neonatal symptom duration was greater than 28 days (the cutoff for classification as a neonatal death), this value was flagged. Next, if the dataset was submitted in multiple sections, we examined the final comprehensive database for any technical issues that may have occurred in merging the individual files. Finally, we merged the dataset with the gold standard medical record information, which was separately transmitted to IHME by the site coordinator. We examined the observations for consistency between the two sources of information, such as the sex of the decedent as reported in the medical record and as reported by the verbal autopsy respondent. Any issues determined through this stringent checking process were compiled into a report and sent to the site to review. Site coordinators were asked to speak with the interview staff and rectify any correctable issues such as data entry mistakes.

### Generation of dichotomized variables

In addition to the full dataset as it was collected, we have also created a series of dichotomous variables from each of the polytomous (categorical) and continuous (duration) variables. Some analytical methods can only use dichotomized variables, so this effort to create the dichotomous variables increases the information available to these types of empirical methods. For each continuous duration item, depending on the item, we identified a short or long cutoff. For example, a duration of 8.8 days marks long duration of a fever. If a VA reports a fever of 10 days, it is considered to have the symptom of "having a long fever." We determine the cutoff as being two median absolute deviations above the median of the mean durations across causes (MAD estimator). The MAD estimator can be used as a robust measure of the standard deviation and is especially useful in cases where extremely long durations may be reported, which would bias measures such as the standard deviation. Additional file 9 shows the cutoffs for each item developed in this way. For polytomous variables, we examined the pattern of the endorsement rates across causes and mapped the categories into two, thus creating a dichotomous version of the variable. For example, we judged that there was a stronger signal produced by combining moderate and severe fevers. Additional file 10 shows the mapping of each response category into dichotomous variables. Based on the data collected, some polytomous variables appeared to have little or no information content and were not mapped into a dichotomous form. These low information content items are shown in Additional file 11. This exercise was undertaken for neonatal, child, and adult modules separately.

### Inclusion of health care experience

There has long been concern that the performance of a VA instrument and the associated analytical method for assigning cause could be different for deaths where the decedent died in a hospital or had made extensive use of health services prior to death, compared to deaths with no health care experience (HCE). As an attempt to examine how VA may work in communities with limited or no access to health care services, Murray et al. [12] studied how PCVA and the Symptom Pattern Method performed when all items referring to use of health services such as "Have you ever been diagnosed with..." or hospital records or death certificates were excluded from the analysis. They showed that, in China, recall of the household or possession of medical records recorded in the VA interview had a profound effect on both the concordance for PCVA as well as the performance of the Symptom Pattern Method.

Given this empirical finding, we believe it is useful to test how excluding household recall of health care experience likely provides a more realistic assessment of how VA performs in communities without access to health services. As such, we have created two versions of the datasets developed above, one version with all variables and one version excluding recall of health care and medical records. Specifically, the without HCE dataset excludes the following information. First, a series of questions asked if the deceased had any specified conditions, which would likely indicate a health care provider had diagnosed the individual. Each of the following conditions was asked: "Did decedent have [asthma, hypertension, obesity, stroke, tuberculosis, AIDS, arthritis, cancer, COPD, dementia, depression, diabetes, epilepsy, heart disease]?" Second, if any medical records were available, the interviewer was asked to provide a transcription of the last note on the medical record. Third, if a death certificate was available, the interviewer was asked to record the immediate cause of death, first underlying cause, second underlying cause, third underlying cause, and contributing causes from the death certificate. Finally, at the end of the questionnaire, an open-ended section was provided to collect any comments from the interviewer, as well as to ask the respondent "to summarize, or tell us in your own words, any additional information about the illness and/or death of your loved one?" Excluding this entire section excludes both open narrative recall of HCE but also, in the case of PCVA, excludes any other information on timing and sequencing of signs and symptoms that might be conveyed in this section.

### Processing free text for use in empirical methods

The structured instrument includes various open text items. First, some questions in the instrument ask the respondent to choose from a list of specified response options. For example, "Where was the rash located?" has the following response options: face, trunk, extremities, everywhere, or "other (specify: ____)." If the response is not one of the listed options, the respondent is asked to fill in the location of the rash as the "other" response. The questions that include an "other" free text response option are as follows: "Where was the rash located?"; "Where was the pain located?"; "Which were the limbs or body parts paralyzed?"; "What kind of tobacco did [NAME] use?"; "Did [NAME] suffer from an injury or accident such as a ____?"; "Where was the deceased born?"; "What were the abnormalities?" in reference to any abnormalities at time of delivery; "Where did the deceased die?"; "What was the color of the liquor when the water broke?" in reference to labor; "Where did the delivery occur?"; and "Who delivered the baby?" In the questions that collect information about a health facility or midwife, free text responses collected the name and address of the place or person. In addition to these free text items, if any medical record or death certificates were available, the interviewer was asked to transcribe the information from the records as free text. Finally, at the end of each interview, the open narrative question "Summarize, or tell us in your own words, any additional information about the illness and/or death of your loved one?"(as described above) was collected in addition to any notes from the interviewer.

Open text could in theory be highly informative, especially household recall of HCE and an interviewer's direct recording of death records or hospital records kept by the household. These observations are likely to be available in populations with some access to health care services. To make this information available to automated methods, we processed open text in the following steps. First, all free text was compiled into a database and a dictionary was created to map all similar words to the same stem word. For example, the terms AMI, myocardial infarction syndrome, acute myocardial infarction, ISHD, MI, coronary heart disease, CHD, IHD, MCI, and MYIN would all be mapped by the dictionary into the same variable ("IHD: Acute Myocardial Infarction"). Next, a program called README [42] extracts each individual variable and assigns a frequency count for the number of times it appears in the entire free text database. Variables that are not deemed to be diagnostically relevant or that are very low in frequency are then dropped from the dataset. The final product is a condensed dictionary of medically important terms consisting of 106 variables for adults, 90 for children, and 39 for neonates. These terms are added as additional binary symptoms (present or not present) in the VA database. If any of the terms appear in the free text for a particular death, it is counted as a positive endorsement for that symptom. These symptoms are not used in the "without" HCE dataset. Additional file 12 provides the comprehensive dictionary that was developed.

### Analysis datasets

**Figure 1:**
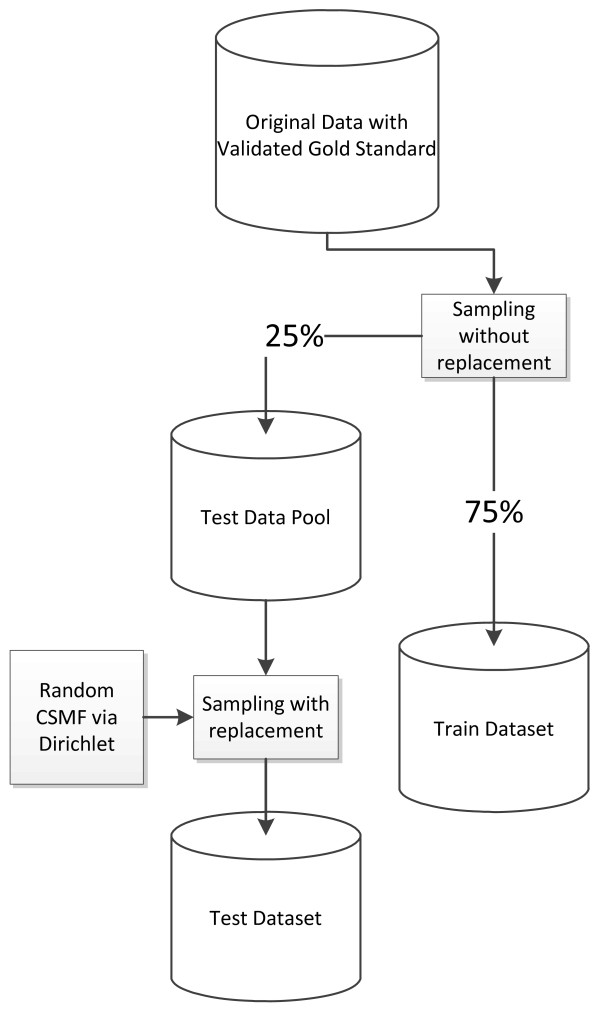
**The process of generating 500 test and training datasets (done separately for each cause of death)**.

For empirical VA methods that must be developed using the pattern of responses observed in a dataset, validation needs to be undertaken on a set of deaths that were not included in the development of the method. This is the concept of a training dataset distinct from a test dataset. Further, as recommended in Murray et al. [15] it is important to have test datasets with widely varying cause-specific mortality fractions (CSMFs) so that a VA method does not by chance appear to be better than another because of the specific CSMF composition in the training set. To facilitate strict comparability, we have created 500 train-test dataset pairs. Each pair was created by first splitting the data randomly (without replacement) into 75%/25% training and test datasets, cause by cause, and then resampling the data in the test dataset (with replacement) to have 7,836 adult, 2,075 child, 1,629 neonatal, and 1,002 stillbirth deaths, matching a cause composition drawn from an uninformative Dirichlet distribution (Figure 1). In other words, each test dataset has been resampled to have a different CSMF composition. Because the CSMF compositions have been drawn from an uninformative Dirichlet, across the 500 test datasets, there are cases where any given cause has a cause fraction near zero and cause fractions as high as 20% or more. By the nature of this sampling strategy, there is no correlation between the CSMF composition of the training and test dataset pairs.

### Shortened cause lists

In order to have an efficient cause list for the analysis, we have reduced it in two steps as illustrated in Table 4. From the original gold standard target cause list we received deaths from the sites for 53 diseases in adults, 27 in children, and 13 in neonates, excluding stillbirths. The first step was to select only those causes with 15 or more deaths (see Additional file 5 for a detailed mapping), and due to that decision we reduced the list into 46 adult causes, 22 child causes, and 12 neonate causes, excluding stillbirths. For instance, pelvic inflammatory diseases, uterine cancer, and dementia in adults; AIDS with tuberculosis in children; and meningitis in neonates had fewer than 15 deaths each. We also eliminated pertussis in children and neonatal tetanus because no pertussis and only four neonatal tetanus deaths were gathered. These deaths were assigned to one of the remaining categories, such as residual categories like "other defined cancers" or "other childhood infectious diseases." In the next step we explored the frequency with which one cause was erroneously classified as another cause in the analysis. For example, deaths due to maternal hemorrhage were often assigned to anemia in the analysis and vice versa. Similarly, all types of diabetes in adults (diabetes with coma, with renal failure, or with skin infection), sepsis with and without local bacterial infection in children, and respiratory distress syndrome in neonates regardless of the gestational age were all frequently hard to differentiate in the analysis. The causes that were frequently confused with each other were aggregated into a new cause in the final analysis cause list. For example, all six maternal causes were combined into one maternal category. After this step, the final cause list for analysis had 34 causes for adults, 21 for children, and 10 for neonates, excluding stillbirths.

**Table 4:** Reduction in number of causes to the final analysis cause list, excluding stillbirths

	Adult	Child	Neonate
**Target cause list**	53	27	13

**>15 deaths**	46	22	12

**Cross classification**	34	21	10

## Results

Table 5 shows that of the 12,542 deaths collected as gold standard cases for the study, the vast majority (88%) were deaths that met the highest level of GS criteria (level 1). This number varies from 84% in Bohol to 91% in Dar es Salaam; and by age, 86% of adult deaths were level 1, 81% of child deaths, and 99.7% of neonate deaths. The majority of the remaining 12% level 2 deaths were adults.

**Table 5:** Numbers of VAs collected by site and gold standard level

Site	Adult		Child		Neonate		Total
	**Level 1**	**Level 2**	**Level 1**	**Level 2**	**Level 1**	**Level 2**	

**Andhra Pradesh**	1,285	269	385	66	376	1	**2,382**

**Bohol**	998	262	234	30	374	0	**1,898**

**Dar es Salaam**	1,556	162	366	106	1,047	2	**3,239**

**Mexico**	1,373	215	124	4	313	2	**2,031**

**Pemba Island**	266	31	156	105	261	3	**822**

**Uttar Pradesh**	1,277	142	412	87	251	1	**2,170**

**Total**	**6,755**	**1,081**	**1,677**	**398**	**2,622**	**9**	**12,542**

It is interesting to note the cause distribution by quality of the gold standards. Table 6 presents the breakdown of how many level 1 and level 2 GS cases were collected for each of the 53 adult causes. Eighty-six percent of adult deaths were level 1, 13% were level 2A, and 1% were level 2B. Twenty five causes of death, which represent 47% of all adult causes, were exclusively level 1. For the remaining 28 causes, the frequency of level 1 deaths varies, such as cirrhosis and asthma with less than 30% level 1 cases; pneumonia and sepsis with between 30% and 60% level 1 cases; and stroke, lung and esophageal cancers, and tuberculosis with between 60% and 75% level 1 cases. Table 7 shows the results for the 2,075 deaths in children. Eighteen causes of death, which comprise 67% of all of the child causes, reached the level 1 gold standard. Another six causes do not achieve more than 60% of gold standard level 1 and vary from 0% (measles) to more than 50% (malaria, pneumonia, and sepsis). Table 8 shows that the level of quality was very high for the 1,629 neonatal deaths and 1,002 stillbirths.

**Table 6:** Numbers of VAs collected by cause of death and gold standard level for adult causes

Adult causes	Level 1	Level 2A	Level 2B
AIDS	345	0	8

AIDS with TB	148	0	0

Acute myocardial infarction	376	24	0

Anemia	68	0	0

Asthma	13	34	0

Bite of venomous animal	66	0	0

Breast cancer	179	3	12

COPD	170	1	0

Cervical cancer	127	23	5

Cirrhosis	82	231	0

Colorectal cancer	85	6	8

Dementia	1	0	0

Diabetes with coma	144	0	0

Diabetes with renal failure	156	0	0

Diabetes with skin infection/sepsis	114	0	0

Diarrhea/dysentery	221	7	0

Drowning	106	0	0

Epilepsy	47	1	0

Esophageal cancer	26	13	1

Falls	173	0	0

Fires	122	0	0

Hemorrhage	111	3	0

Homicide	167	0	0

Hypertensive disorder	107	6	0

Congestive heart failure	221	0	0

Inflammatory heart disease	42	0	0

Leukemia	71	2	5

Liver cancer	29	0	2

Lung cancer	66	36	4

Lymphomas	74	0	3

Malaria	89	11	0

Mouth/oropharynx cancer	22	0	0

Obstructed labor	17	1	0

Other cancers	142	0	0

Other cardiovascular diseases	153	0	0

Other digestive diseases	166	0	0

Other infectious diseases	258	0	0

Other injuries	103	0	0

Other noncommunicable diseases	200	0	0

Other pregnancy-related deaths	89	0	0

Ovarian cancer	32	1	0

Pelvic inflammatory disease	5	0	0

Pneumonia	310	229	0

Poisonings	86	0	0

Prostate cancer	40	8	0

Renal failure	411	2	0

Road traffic	202	0	0

Sepsis	24	46	0

Stomach cancer	50	10	2

Stroke	378	252	0

Suicide	124	0	0

TB	196	79	0

Uterine cancer	1	1	1

**Table 7:** Numbers of VAs collected by cause of death and gold standard level for child causes

Child causes	Level 1	Level 2A	Level 2B
AIDS	19	0	0

AIDS with TB	1	0	0

Bite of venomous animal	54	0	0

Diarrhea/dysentery	255	1	0

Drowning	82	1	0

Encephalitis	41	0	0

Falls	49	0	0

Fires	68	0	0

Hemorrhagic fever	51	0	0

Malaria	59	58	0

Measles	0	23	0

Meningitis	58	0	0

Other cancers	28	0	0

Other cardiovascular diseases	76	0	0

Other defined causes of child deaths	182	0	0

Other digestive diseases	48	0	0

Other infectious diseases	60	0	0

Other respiratory diseases	12	0	0

Pertussis	0	0	0

Pneumonia	272	224	1

Pneumonia and diarrhea	35	3	0

Poisonings	18	0	0

Road traffic	92	0	0

Sepsis (with local bacterial infection)	22	15	0

Sepsis (without local bacterial infection)	39	67	0

TB	4	5	0

Violent death	52	0	0

**Table 8:** Numbers of VAs collected by cause of death and gold standard level for neonatal causes

Neonate causes	Level 1	Level 2A	Level 2B
Birth asphyxia	461	0	0

Congenital malformation	250	0	0

Meningitis (serious infection)	6	0	0

Pneumonia (serious infection)	84	5	0

Preterm delivery (<33 weeks gestational age [GA]) without respiratory distress syndrome (RDS)	353	0	0

Preterm delivery (with or without RDS) and sepsis	75	1	0

Preterm delivery (without RDS) and birth asphyxia	89	0	0

Preterm delivery (without RDS) and sepsis and birth asphyxia	34	0	0

Respiratory distress syndrome (33-36 weeks GA)	13	0	0

Respiratory distress syndrome (<33 weeks GA)	97	0	0

Sepsis (serious infection)	127	1	0

Sepsis with local bacterial infection	32	1	0

Stillbirth	1,001	1	0

Tetanus	4	0	0

The distribution of cases (all criteria levels combined) across the six sites is shown in Additional file 13. The relative distribution of cases by age of death across sites reflects their overall progress with mortality transition. Thus adult deaths were comparatively fewer in Pemba compared to all other sites where 1,200 to 1,600 cases were typically collected. Larger numbers of child deaths were collected in Dar es Salaam and Uttar Pradesh, where child death rates are higher than elsewhere. Similar numbers of neonatal deaths were collected in each site (250 to 400) except for Dar es Salaam. In this case, the site collected VAs on a significantly higher number of neonatal deaths (1049) than was targeted, as the site had the VA interviewer capacity to easily add these cases as they were identified. For example, while the targeted number of stillbirth deaths was 100, the Dar es Salaam site was able to easily collect interviews on 432 cases to help build a more robust dataset.

## Discussion

PHMRC was able to obtain completed VA interviews for more than 12,000 deaths with GS assignment of true cause of death. Because of the poor quality of medical record-keeping and limitations of diagnostic technology in many hospitals, to identify more than 12,000 GS deaths required reviewing and screening a much larger number of records. While it was difficult in many sites to obtain sufficient documentation for some causes of death overall across all six sites, we were able to find enough deaths for 46 adult causes, 22 child causes, and 12 neonate causes, excluding stillbirths, from the original cause list. The implementation of the project revealed just how poor the quality of medical records and diagnosis is in some institutions. This finding reaffirms our original hypothesis that convergent validity between verbal autopsy and poorly assigned hospital cause of death is not a measure of criterion validity.

An important potential limitation of the study is the extent to which the cause of death based on fulfilling the clinical, laboratory, medical imaging, and tissue pathology criteria in this study are the true cause of death. Studies in high-resource settings [43] suggest that clinical diagnosis compared to postmortem autopsy may differ in up to 25% of cases. These studies, however, exaggerate the limitations of our study using clinical diagnostic criteria for three reasons. First, autopsies are much more likely to be undertaken in medico-legal cases or cases with uncertain clinical diagnosis. Shojania et al. found that once the inherent selection bias of postmortem autopsy is taken into account, clinical diagnosis and postmortem autopsy agree more than 90% of the time [44]. Second, these comparisons are for all clinical diagnoses, not for the subset that meets our clearly defined and stringent criteria. In general, less than one-third of hospital deaths in our study fulfilled our diagnostic criteria even in the most sophisticated hospitals. It is a reasonable assumption that the concordance between the clinical diagnosis and postmortem autopsy would be even higher in the subset meeting our criteria. Finally, the definition in these studies of major diagnostic discrepancy is for clinical purposes, not for the purposes of assigning underlying cause of death. For the latter effort, some of the major discrepancies would not move deaths between cause of death categories used in this study.

Some readers may object to the use of "gold standard" in describing our dataset. We believe, however, that we have implemented the best possible approach to assigning causes of death. In nearly all settings, postmortem rates are low and subject to severe selection bias toward diagnostically challenging and nonrepresentative deaths for a cause. For both implementation and selection bias reasons, we do not foresee VA validation studies being undertaken using large samples of deaths with postmortem autopsies. Clearly defined clinical, laboratory, imaging, and tissue pathology criteria as used in this study are the best that can be implemented. As such, we believe the use of the term gold standard for this dataset is appropriate.

A particularly vexing issue in VA validation studies is that by their nature they are conducted on deaths that have occurred in hospital. What would be the performance of VA for deaths in the community? There are potentially three distinct aspects to this question. First, the cause-composition of deaths in the hospital and the community will be different. Fortunately, because we create multiple test datasets with widely varying cause compositions, this issue will not influence the results from VA validation studies as long as the methods recommended by Murray et al. [15] are followed. Second, contact and experience with the health system could change the way in which household members recall certain symptoms or signs. If it does, then VA may capture more information in those cases with hospital experience than when implemented in a population with little or no experience of health care. Given that all validation studies require some diagnostic information on the course of illness prior to death, no validation study can ever investigate this question. This is an unfortunate reality; we believe that constructing a dataset, as we have done, that excludes all information from the household about medical experience prior to death is the closest we can come in a validation study to understanding how VA will perform in a poor, underserved community. While it is theoretically possible that household recall of symptoms and signs will be different if someone has experienced health care prior to death, there is in fact no direct evidence for this hypothesis, nor is it clear how it would be tested. Third, the clinical course and thus the signs and symptoms related to a cause of death may be influenced through contact with the health system. As with the second limitation, there is unfortunately no way to investigate this important issue. We simply have no way to figure out the true cause of death for deaths that have occurred in the community with no contact with health services.

Ideally, all countries would have in place functioning vital registration systems that capture all deaths and include a medically certified cause of death according to the procedures and rules of the International Classification of Diseases in force at the time. While progress toward this goal is being made, it is painfully slow, and without greater government commitment, will not be a reality for most developing countries for decades to come [45,46]. To meet urgent policy and planning needs, countries will have no alternative but to introduce verbal autopsy, at least for deaths that occur outside hospitals. It is critically important that they have confidence in the VA methods they use, and that they understand the validation and performance characteristics of those methods. We believe that to do so, validity and comparative performance must be assessed against rigorous, standardized criteria that unambiguously identify the cause of death, and that are not influenced whatsoever by the quality, usually very poor, of medical records or the diagnostic biases of physicians who review them. Our study has compiled the first ever dataset of gold standard cause of death assignments across six sites in four countries. It is unlikely that a comparable dataset on VA with true gold standard cause of death ascertainment will be collected in the near future, if for no other reason than the substantial cost and time investment. For quite some time, therefore, the PHMRC will be the largest and most rigorously collected VA validation set. We intend to make the dataset publicly available in the hope that it will serve as a resource for the broader VA scientific community interested in developing and testing new methods. For this reason, we plan to release to the public an anonymized version of the dataset once the primary set of analyses from the investigators have been published.

One lesson learned from the complexity of converting free text into dichotomous variables is that future VA instruments may want to incorporate a series of checklist questions based on the free text variables that improve VA performance. Rather than free text, items could be included such as "Did anyone tell you or do you have any documentation mentioning acute myocardial infarction, MI, ischemic heart disease, or coronary heart disease?" These checklist items would be completed by the interviewer after questioning the respondent and examining the medical records and other documentation available. In this way, the task of reading free text and translating it through a dictionary would be simplified and focused only where it is likely to change the results.

## Conclusion

We have described the development and usefulness of the largest, perhaps only dataset with gold standard cause of death assignment and matching verbal autopsies for more than 12,000 deaths in four countries. We expect that this will facilitate further development of verbal autopsy and perhaps other cause of death measurement approaches in countries with poor vital registration and certification practices. The utility of this dataset will undoubtedly improve if additional cases, in different populations, and for different diseases than those reported here, are added in future studies, provided the same protocols and standards are applied. In this way, confidence in the utility of verbal autopsy methods will increase and result in their wider application in countries to reduce ignorance about the comparative importance of leading causes of death.

## Abbreviations

CSMF: cause-specific mortality fractions; GS: gold standard; HCE: health care experience; MAD: median absolute deviation; MDEF: medical data extraction form; PCVA: physician-certified verbal autopsy; PHMRC: Population Health Metrics Research Consortium; VA: verbal autopsy; WHO: World Health Organization

## Competing interests

The authors declare that they have no competing interests.

## Authors' contributions

CJLM, ADL, and RB conceptualized and organized the study. CJLM, ADL, RL, and SLO drafted the manuscript. DP, RJ, and BN directed the data collection at the Andhra Pradesh site; IR, ML, DS, VT, and HR directed the data collection at the Bohol site; ZP, ED, WF, SM, and MS directed the data collection at the Dar es Salaam site; BH, SG, RL, DRV, and MR directed the data collection at the Mexico site; SS, SMA, UD, and AD directed the data collection at the Pemba site; and VK, RA, VD, AK, and RP directed the data collection at the Uttar Pradesh site. AB, LD, ADF, HK, RL, and IR conceptualized analytic strategies and development of methods. SLO organized and managed collaboration, data collection, and analytics. All authors have read and approved the final manuscript.

## Supplementary Material

Additional file 1 **Differences between the standardized WHO instrument and the PHMRC instrument**. Click here for file

Additional file 2 **General module of the full verbal autopsy instrument used in the field in the PHMRC study**. Click here for file

Additional file 3 **Adult module of the full verbal autopsy instrument used in the field in the PHMRC study**. Click here for file

Additional file 4 **Child and neonate module of the full verbal autopsy instrument used in the field in the PHMRC study**. Click here for file

Additional file 5 **Target cause list reduced to analysis cause list**. Click here for file

Additional file 6 **Gold standard (GS) definitions in the PHMRC study for adults**. Click here for file

Additional file 7 **Gold standard (GS) definitions in the PHMRC study for children/neonates**. Click here for file

Additional file 8 **Medical data extraction form (MDEF) used to extract gold standard data for the PHMRC study**. Click here for file

Additional file 9 **Duration cutoffs used when making variables dichotomous**. Click here for file

Additional file 10 **Conversion of polytomous symptoms into dichotomous symptoms**. Click here for file

Additional file 11 **Low-content polytomous items including response frequencies**. Click here for file

Additional file 12 **README dictionary developed to convert free text responses into usable key terms**. Click here for file

Additional file 13 **Final analysis cause list and numbers of deaths by site**. Click here for file
