# Supplementary material for: Population Health Metrics Research Consortium gold standard verbal autopsy validation study: design, implementation, and development of analysis datasets
Source: Popul Health Metr. 2011 Aug 4;9:27. doi: 10.1186/1478-7954-9-27 (PMC3160920; doi:10.1186/1478-7954-9-27)
Supplement: Additional file 2 — General module of the full verbal autopsy instrument used in the field in the PHMRC study. [file 1478-7954-9-27-S2.DOC]

**POPULATION HEALTH METRICS RESEARCH CONSORTIUM VERBAL AUTOPSY INSTRUMENT**

**GENERAL INFORMATION MODULE**

**BEFORE THE INTERVIEW, FILL IN THIS SECTION FROM THE MEDICAL RECORDS OF THE DECEASED.**

**SECTION 1: BACKGROUND ABOUT DECEASED**

| 1.1 | Date of birth (if known) | _ _ / _ _ / _ _ _ _  dd / mm / yyyy | |
| --- | --- | --- | --- |
| 1.2 | Name of deceased (if known) |  | |
| 1.3 | Name of mother (if deceased <12 yrs) |  | |
| 1.4 | Name of father (if deceased <12 yrs) |  | |
| 1.5 | Sex of deceased | 1. Male 2. Female |    |
| 1.6 | Date of Death | _ _ / _ _ / _ _ _ _  dd / mm / yyyy |  |
| 1.7 | Last known age of the deceased  *(Record “00” days if less than one day)*  *(99 = “Don’t know”)* | ____ ____years (if one year or older)  ____ ____ months (if less than one year)  ____ ____ days (if less than one month) |  |
|  | *If deceased is less than 12 years of age, then skip to 1.9.* | | |
| 1.8 | Marital status of deceased | 1. Never married 2. Married 3. Separated 4. Divorced 5. Widowed |          |
| 1.9 | Last known level of education attended by the deceased.    *Each site should modify this question if needed for their site.* | 1. No education 2. Primary school 3. Secondary school 4. Post secondary 5. Not listed |          |
|  |  |  |  |
| 1.10 | Number of years completed | ___ ____ years  9. Don’t know |  |
|  |  |  |  |

**BEFORE THE INTERVIEW, FILL IN THIS SECTION. THESE QUESTIONS SHOULD NOT BE ASKED TO THE RESPONDENT.**

**SECTION 2: BACKGROUND ABOUT INTERVIEW**

|  |  |  | |  | |
| --- | --- | --- | --- | --- | --- |
| 2.1 | Language of Interview |  | |  | |
| 2.2 | Interviewer name and/or ID number (fill in as needed) | ID Number __ __ __ __ __ | | | |
| 2.3 | Date of attempted and successful interviews: |  | |  | |
|  | Date of first interview attempt | _ _ / _ _ / _ _ _ _  dd / mm / yyyy | | | |
|  | Date and time arranged for second interview attempt | _ _ / _ _ / _ _ _ _  dd / mm / yyyy  ________ (Time) | | | |
|  | Date and time arranged for third interview attempt | _ _ / _ _ / _ _ _ _  dd / mm / yyyy  ________ (Time) | | | |
|  | Date of interview | _ _ / _ _ / _ _ _ _  dd / mm / yyyy | | | |
|  | Date form checked by supervisor | _ _ / _ _ / _ _ _ _  dd / mm / yyyy | | | |
|  | Date entered in computer | _ _ / _ _ / _ _ _ _  dd / mm / yyyy | | | |
|  | | | | | |
| 2.4 | Address of/directions to household |  | |  | |
|  |  |  | |  | |
|  |  |  | |  | |
|  |  |  | |  | |
|  |  |  | |  | |
|  |  |  | |  | |
|  |  |  | |  | |
|  |  |  | |  | |
| 2.5 | Sketch a Map if Needed | |  | |  |
|  | | | | | |

**INTERVIEW BEGINS**

*Instructions to interviewer: Introduce yourself and explain the purpose of your visit. Ask to speak to the mother or to another adult who was the deceased’s main caretaker during the illness that led to death. If this is not possible, arrange a time to revisit the household when the caretaker will be home. (see example below).*

“My name is [your name]. I am an interviewer with the Population Health Metrics Research Consortium project. I have been informed that a death has occurred in your household. I am very sorry to hear that a member of your household has passed away. Please accept my sympathies. For the purpose of improving health care, we are collecting information on all recent deaths in this area. I would like to talk to the mother or main caretaker of [the deceased’s name] and ask some questions about the events and any symptoms that [the deceased’s name] had during her/his illness before death.”

**SECTION 3: CONSENT**

|  | ***INTERVIEWER: Read the consent form to the respondent. Ask the respondent if he or she has any questions. Once any questions are answered, ask the respondent if he or she is willing to take part in the study.*** | |  |
| --- | --- | --- | --- |
|  |  |  |  |
| 3.1 | *Did respondent give consent?* | 1. Yes 2. No |    |
|  | ***If answer is “Yes” proceed to 4.1.***  ***If answer is “No” then thank respondent for their time and end the interview.*** |  |  |

**SECTION 4: INFORMATION ABOUT RESPONDENT**

|  | *Instructions to Interviewer: Inform the respondent that you would now like to ask him/her some general questions about himself/herself.* | |  |
| --- | --- | --- | --- |
| 4.1 | What is your (the respondent’s) name? |  |  |
| 4.2 | *INTERVIEWER: What is the sex of the respondent?* | 1. Male  2. Female |    |
| 4.3 | What is your (the respondent’s) relationship to the deceased? (*mark relevant box*) | 1. Mother  2. Father  3. Grandmother  4. Grandfather  5. Aunt  6. Uncle  7. Husband  8. Wife  9. Brother  10. Sister  11. Birth attendant  (type _________)  12. Other male  (specify _______)  13. Other female  (specify_______) |                            |
| 4.4 | How old are you? | __ __ years |  |
| 4.5 | What was the last level of school you attended?  *Each site should modify this question if needed for their site.* | 1. No education  2. Primary school  3.Secondary school  4. Post secondary  5. Not listed |          |
|  | *Instructions to Interviewer: Tell the respondent:*  *“I would now like to ask you some questions about your household. Please remember that all information will be kept confidential.”* | |  |

| 4.6 | How many people live at this address? | __ __ |  |
| --- | --- | --- | --- |
| 4.7 | How many rooms are in the household? | __ __ |  |
| 4.8 | Does the household have a separate room for cooking? | 1. Yes 2. No |    |

| 4.9 | Does the household have:  *(Mark all the apply)* | 1. Electricity  2. A radio  3. A television  4. A refrigerator  5. A fixed line telephone  6. A mobile telephone  7. A computer  8. A bicycle  9. A car or truck  10. Piped water outside the residence  11. Well (protected or unprotected)  12. Water vendor, water supplied by truck, or bottled water  13. Surface water | Yes   No   Yes   No   Yes   No   Yes   No   Yes   No   Yes   No   Yes   No   Yes   No   Yes   No   Yes   No   Yes   No   Yes   No   Yes   No  |
| --- | --- | --- | --- |
| 4.10 | What type of toilet do you have/use? | 1. Flush toilet  2. Improved pit toilet  3. Traditional pit toilet  4. Bush/field/beach  5. Other  (specify)­­­­__________ | Yes   No   Yes   No   Yes   No   Yes   No   Yes   No  |
| 4.11 | What is the main kind of energy you use for cooking? | 1. Charcoal  2. Firewood  3. Kerosene  4. Electricity  5. Gas  6. Cow dung  7. Other  (specify)___________ | Yes   No   Yes   No   Yes   No   Yes   No   Yes   No   Yes   No   Yes   No  |
|  | *INTERVIEWER: What materials are used for the floor of the house?*  *(Mark as observed)* | 1. Natural/mud  2. Cement  3. Wood  4. Tiles  5. Others  (specify)__________ | Yes   No   Yes   No   Yes   No   Yes   No   Yes   No  |

**SECTION 5: QUESTIONS ON THE DECEASED**

| 5.1 | When was the deceased born? | _ _ / _ _ / _ _ _ _  dd / mm / yyyy |  |
| --- | --- | --- | --- |
| 5.2 | What was the sex of the deceased? | 1. Male  2. Female |    |
| 5.3 | When did he/she die? | _ _ / _ _ / _ _ _ _  dd / mm / yyyy |  |
| 5.4 | What was the last known age of the deceased?  *Less than 24 hours = 00 days.* | ___ ___ years (if one year or older)  ___ ___ months (if less than one year)  ___ __ days (if less than one month) |  |
|  | *If he/she is less than 12 years of age, then skip to 5.6.*  *If he/she is less than 5 years of age, then skip to 5.7.* |  |  |
| 5.5 | What was the marital status of deceased? | 1. Never married  2. Married  3. Separated  4. Divorced  5. Widowed |          |
| 5.6 | What was the last known level of education that he/she attended? | 1. No education  2. Primary school  3.Secondary school  4. Post secondary  5. Not listed |  |

| 5.7 | *INTERVIEWIER: Are there other people present during the interview?* | | | | 1. Yes 2. No |    |
| --- | --- | --- | --- | --- | --- | --- |
|  | *If “Yes”, provide the below information for any additional respondents. If “No” go to 5.10.* | | | |  |  |
| 5.8 | *How many people are present during the interview?* | | | | __ __ |  |
| 5.9 | *INTERVIEWER: For each person present during the interview, determine their relationship to the deceased and mark whether the person was present during the illness and/or present at death.* | | | | |  |
|  | |  | Present During Illness | Present at Death | | |
|  | | Mother | 1. Yes   2. No  | 1. Yes   2. No  | | |
|  | | Father | 1. Yes   2. No  | 1. Yes   2. No  | | |
|  | | Aunt | 1. Yes   2. No  | 1. Yes   2. No  | | |
|  | | Uncle | 1. Yes   2. No  | 1. Yes   2. No  | | |
|  | | Grandmother | 1. Yes   2. No  | 1. Yes   2. No  | | |
|  | | Grandfather | 1. Yes   2. No  | 1. Yes   2. No  | | |
|  | | Husband | 1. Yes   2. No  | 1. Yes   2. No  | | |
|  | | Wife | 1. Yes   2. No  | 1. Yes   2. No  | | |
|  | | Brother | 1. Yes   2. No  | 1. Yes   2. No  | | |
|  | | Sister | 1. Yes   2. No  | 1. Yes   2. No  | | |
|  | | Traditional Birth Attendant | 1. Yes   2. No  | 1. Yes   2. No  | | |
|  | | Other Male (specify) | 1. Yes   2. No  | 1. Yes   2. No  | | |
|  | | Other Female (specify) | 1. Yes   2. No  | 1. Yes   2. No  | | |

5.10

| **STOP.**  **If deceased was less than 12 years old, begin Neonatal and Child VA module. If deceased was 12 years or older, begin Adult VA module.** |
| --- |
