# Supplementary material for: Population Health Metrics Research Consortium gold standard verbal autopsy validation study: design, implementation, and development of analysis datasets
Source: Popul Health Metr. 2011 Aug 4;9:27. doi: 10.1186/1478-7954-9-27 (PMC3160920; doi:10.1186/1478-7954-9-27)
Supplement: Additional file 3 — Adult module of the full verbal autopsy instrument used in the field in the PHMRC study. [file 1478-7954-9-27-S3.DOC]

**POPULATION HEALTH METRICS RESEARCH CONSORTIUM**

**ADULT AND ADOLESCENT VERBAL AUTOPSY MODULE**

**SECTION 1: HISTORY OF CHRONIC CONDITIONS OF THE DECEASED**

| 1.1 | Did the deceased have any of the following? |  |  |
| --- | --- | --- | --- |
|  | Asthma | 1. Yes  2. No  8. Refused to answer  9. Don’t know | ****  ****  ****  **** |
|  | Arthritis | 1. Yes  2. No  8. Refused to answer  9. Don’t know | ****  ****  ****  **** |
|  | Cancer | 1. Yes  2. No  8. Refused to answer  9. Don’t know | ****  ****  ****  **** |
|  | Tuberculosis | 1. Yes  2. No  8. Refused to answer  9. Don’t know | ****  ****  ****  **** |
|  | Dementia | 1. Yes  2. No  8. Refused to answer  9. Don’t know | ****  ****  ****  **** |
|  | Depression | 1. Yes  2. No  8. Refused to answer  9. Don’t know | ****  ****  ****  **** |
|  | Diabetes | 1. Yes  2. No  8. Refused to answer  9. Don’t know | ****  ****  ****  **** |
|  | Epilepsy | 1. Yes  2. No  8. Refused to answer  9. Don’t know | ****  ****  ****  **** |
|  | Heart Disease | 1. Yes  2. No  8. Refused to answer  9. Don’t know | ****  ****  ****  **** |
|  | High Blood Pressure | 1. Yes  2. No  8. Refused to answer  9. Don’t know | ****  ****  ****  **** |
|  | Obesity | 1. Yes  2. No  8. Refused to answer  9. Don’t know | ****  ****  ****  **** |
|  | Stroke | 1. Yes  2. No  8. Refused to answer  9. Don’t know | ****  ****  ****  **** |
|  | COPD (Chronic Obstructive Pulmonary Disease) | 1. Yes  2. No  8. Refused to answer  9. Don’t know | ****  ****  ****  **** |
|  | AIDS | 1. Yes  2. No  8. Refused to answer  9. Don’t know | ****  ****  ****  **** |

**SECTION 2: SYMPTOM CHECKLIST**

| 2.1 | For how long was ______ ill before s/he died? | 1. Months ___ ___  2. Days ___ ____  8. Refused to answer  9. Don’t know | ****  **** |
| --- | --- | --- | --- |

| 2.2 | Did _____ have a fever? | 1. Yes  2. No  8. Refused to answer  9. Don’t know | ****  ****  ****  **** |
| --- | --- | --- | --- |

*If “No” or “Refused to answer” or “Don’t know” go to question 2.7.*

| 2.3 | How many days did the fever last? | Days ___ ____  8. Refused to answer  9. Don’t know | ****  **** |
| --- | --- | --- | --- |
| 2.4 | How severe was the fever? | 1. Mild  2. Moderate  3. Severe  8. Refused to answer  9. Don’t know | ****  ****  ****  ****  **** |
| 2.5 | What was the pattern of fever? | 1. Continuous  2. On and off  3. Only at night  8. Refused to answer  9. Don’t know | ****  ****  ****  ****  **** |
| 2.6 | Did ______ have sweating with the fever? | 1. Yes  2. No  8. Refused to answer  9. Don’t know | ****  ****  ****  **** |

| 2.7 | Did _____ have a rash? | 1. Yes  2. No  8. Refused to answer  9. Don’t know | ****  ****  ****  **** |
| --- | --- | --- | --- |

*If “No” or “Refused to answer” or “Don’t know” go to question 2.10.*

| 2.8 | How many days did ______ have the rash? | Days ___ ____  8. Refused to answer  9. Don’t know | ****  **** | |
| --- | --- | --- | --- | --- |
| 2.9 | Where was the rash located? | 1. Face  2. Trunk  3. Extremities  4. Everywhere  5, Other (specify)  ________________  8. Refused to answer  9. Don’t know | ****  ****  ****  ****  ****  ****  **** |  |
| 2.10 | Did ____ have sores? | 1. Yes  2. No  8. Refused to answer  9. Don’t know | ****  ****  ****  **** |  |

*If “No” or “Refused to answer” or “Don’t know” go to question 2.12.*

| 2.11 | Did the sores have clear fluid or pus? | 1. Yes  2. No  8. Refused to answer  9. Don’t know | ****  ****  ****  **** |
| --- | --- | --- | --- |
| 2.12 | Did ______ have itching of skin? | 1. Yes  2. No  8. Refused to answer  9. Don’t know | ****  ****  ****  **** |
| 2.13 | Did ______ have an ulcer (pit) on the foot? | 1. Yes  2. No  8. Refused to answer  9. Don’t know | ****  ****  ****  **** |

*If “No” or “Refused to answer” or “Don’t know” go to question 2.16.*

| 2.14 | Did the ulcer ooze pus? | 1. Yes  2. No  8. Refused to answer  9. Don’t know | ****  ****  ****  **** |
| --- | --- | --- | --- |

*If “No” or “Refused to answer” or “Don’t know” go to question 2.16.*

| 2.15 | For how many days did the ulcer ooze pus? | Days ___ ____  8. Refused to answer  9. Don’t know | ****  **** |
| --- | --- | --- | --- |
| 2.16 | Did _______ experience “pins and needles” in their feet? | 1. Yes  2. No  8. Refused to answer  9. Don’t know | ****  ****  ****  **** |
| 2.17 | Did _______ have blue lips? | 1. Yes  2. No  8. Refused to answer  9. Don’t know | ****  ****  ****  **** |

| 2.18 | Had ______ lost weight in the three months prior to death? | 1. Yes  2. No  8. Refused to answer  9. Don’t know | ****  ****  ****  **** |
| --- | --- | --- | --- |

*If “No” or “Refused to answer” or “Don’t know” go to question 2.20.*

| 2.19 | How substantial was the loss of weight? | 1. Slight  2. Moderate  3. Large  8. Refused to answer  9. Don’t know | ****  ****  ****  ****  **** |
| --- | --- | --- | --- |

| 2.20 | Did _____ look pale? | 1. Yes  2. No  8. Refused to answer  9. Don’t know | ****  ****  ****  **** |
| --- | --- | --- | --- |

| 2.21 | Did _____ have yellow discoloration of the eyes? | 1. Yes  2. No  8. Refused to answer  9. Don’t know | ****  ****  ****  **** |
| --- | --- | --- | --- |

*If “No” or “Refused to answer” or “Don’t know” go to question 2.23.*

| 2.22 | For how long did ______ have the yellow discoloration? | 1. Months ___ ___  2. Days ___ ____  8. Refused to answer  9. Don’t know | ****  **** |
| --- | --- | --- | --- |

| 2.23 | Did _____ have ankle swelling? | 1. Yes  2. No  8. Refused to answer  9. Don’t know | ****  ****  ****  **** |
| --- | --- | --- | --- |

*If “No” or “Refused to answer” or “Don’t know” go to question 2.25.*

| 2.24 | For how long did ______ have ankle swelling? | 1. Months ___ ___  2. Days ___ ____  8. Refused to answer  9. Don’t know | ****  **** |  |  |
| --- | --- | --- | --- | --- | --- |

| 2.25 | Did _____ have puffiness of the face? | 1. Yes  2. No  8. Refused to answer  9. Don’t know | ****  ****  ****  **** |
| --- | --- | --- | --- |
|  |  |  |  |

*If “No” or “Refused to answer” or “Don’t know” go to question 2.27*

| 2.26 | For how long did ______ have puffiness of the face? | 1. Months ___ ____  2. Days ____ _____  8. Refused to answer  9. Don’t know | ****  **** |
| --- | --- | --- | --- |
| 2.27 | Did _______ have general puffiness all over his/her body? | 1. Yes  2. No  8. Refused to answer  9. Don’t know | ****  ****  ****  **** |
|  | *If “No” or “Refused to answer” or “Don’t know” go to question 2.29.* | | |
| 2.28 | For how long did _______ have puffiness all over his/her body? | Months ___ ____  Days ____ _____  8. Refused to answer  9. Don’t know | ****  **** |

| 2.29 | Did _____ have a lump in the neck? | 1. Yes  2. No  8. Refused to answer  9. Don’t know | ****  ****  ****  **** |
| --- | --- | --- | --- |
| 2.30 | Did _____ have a lump in the armpit? | 1. Yes  2. No  8. Refused to answer  9. Don’t know | ****  ****  ****  **** |
| 2.31 | Did _____ have a lump in the groin? | 1. Yes  2. No  8. Refused to answer  9. Don’t know | ****  ****  ****  **** |

| 2.32 | Did _____ have a cough? | 1. Yes  2. No  8. Refused to answer  9. Don’t know | ****  ****  ****  **** |
| --- | --- | --- | --- |

*If “No” or “Refused to answer” or “Don’t know” go to question 2.36.*

| 2.33 | For how long did ______ have a cough? | 1. Months ___ ____  2. Days ____ ____  8. Refused to answer  9. Don’t know | ****  **** |
| --- | --- | --- | --- |
| 2.34 | Did the cough produce sputum? | 1. Yes  2. No  8. Refused to answer  9. Don’t know | ****  ****  ****  **** |
| 2.35 | Did _____ cough blood? | 1. Yes  2. No  8. Refused to answer  9. Don’t know | ****  ****  ****  **** |

| 2.36 | Did _____ have difficulty breathing? | 1. Yes  2. No  8. Refused to answer  9. Don’t know | ****  ****  ****  **** |
| --- | --- | --- | --- |

*If “No” or “Refused to answer” or “Don’t know” go to question 2.40.*

| 2.37 | For how long did ____ have difficulty breathing? | 1. Months ___ ____  2. Days ____ ____  8. Refused to answer  9. Don’t know | ****  **** |
| --- | --- | --- | --- |
| 2.38 | Was the difficulty continuous or on and off? | 1. Continuous  2. On and off  8. Refused to answer  9. Don’t know | ****  ****  ****  **** |
| 2.39 | In what position did the difficulty get worse?  *(Read each choice in sequence)* | 1. Lying  2. Sitting  3. Walking/During exertion  4. Didn’t matter  8. Refused to answer  9. Don’t know | ****  ****  ****  ****  **** |

| 2.40 | Did _____ have fast breathing? | 1. Yes  2. No  8. Refused to answer  9. Don’t know | ****  ****  ****  **** |  |  |
| --- | --- | --- | --- | --- | --- |

*If “No” or “Refused to answer” or “Don’t know” go to question 2.42.*

| 2.41 | For how long did ______ have fast breathing? | 1. Months ___ ____  2. Days ____ ____  8. Refused to answer  9. Don’t know | ****  **** |
| --- | --- | --- | --- |
| 2.42 | Did _______ wheeze?  *(Demonstrate)* | 1. Yes  2. No  8. Refused to answer  9. Don’t know | ****  ****  ****  **** |

| 2.43 | Did _____ experience pain in the chest in the month preceding death? | 1. Yes  2. No  8. Refused to answer  9. Don’t know | ****  ****  ****  **** |  |  |
| --- | --- | --- | --- | --- | --- |

*If “No” or “Refused to answer” or “Don’t know” go to question 2.47.*

| 2.44 | How long did the pain last? | 1. Less than  30 minutes  2. 30 minutes to  24 hours  3. More that 24 hours  8. Refused to answer  9. Don’t know | ****  ****  ****  ****  **** |
| --- | --- | --- | --- |
| 2.45 | Was the pain during physical activity? | 1. Yes  2. No  8. Refused to answer  9. Don’t know | ****  ****  ****  **** |
| 2.46 | Where was the pain located?  *(Read each choice in sequence.)* | 1. Upper or middle chest  2. Lower chest  3. Left arm  4. Other (Specify: _______________)  8. Refused to answer  9. Don’t know | ****  ****  ****  ****  ****  **** |

| 2.47 | Did _____ have more frequent loose or liquid stools than usual? | 1. Yes  2. No  8. Refused to answer  9. Don’t know | ****  ****  ****  **** |
| --- | --- | --- | --- |

*If “No” or “Refused to answer” or “Don’t know” go to question 2.49.*

| 2.48 | For how long before death did _____ have loose or liquid stools? | 1. Days ____ _____  8. Refused to answer  9. Don’t know | ****  **** |
| --- | --- | --- | --- |

| 2.49 | Did _____ have a change in bowel habits? | 1. Yes  2. No  8. Refused to answer  9. Don’t know | ****  ****  ****  **** |  |
| --- | --- | --- | --- | --- |
| 2.50 | Was there blood in the stool? | 1. Yes  2. No  8. Refused to answer  9. Don’t know | ****  ****  ****  **** |  |

*If “No” or “Refused to answer” or “Don’t know” go to question 2.52.*

| 2.51 | Was there blood in the stool up until death? | 1. Yes  2. No  8. Refused to answer  9. Don’t know | ****  ****  ****  **** |
| --- | --- | --- | --- |

| 2.52 | Did _________ stop urinating? | 1. Yes  2. No  8. Refused to answer  9. Don’t know | ****  ****  ****  **** |
| --- | --- | --- | --- |

| 2.53 | Did _____ vomit in the week preceding the death? | 1. Yes  2. No  8. Refused to answer  9. Don’t know | ****  ****  ****  **** |
| --- | --- | --- | --- |

*If “No” or “Refused to answer” or “Don’t know” go to question 2.57.*

| 2.54 | For how long before death did ______ vomit? | 1. Days ___ ___  2. Hours ___ ___  8. Refused to answer  9. Don’t know | ****  **** | |
| --- | --- | --- | --- | --- |
|  |  |  |  | |
| 2.55 | Was there blood in the vomit? | 1. Yes  2. No  8. Refused to answer  9. Don’t know | ****  ****  ****  **** |  |
| 2.56 | Was the vomit black? | 1. Yes  2. No  8. Refused to answer  9. Don’t know | ****  ****  ****  **** |  |

| 2.57 | Did _____ have difficulty swallowing? | 1. Yes  2. No  8. Refused to answer  9. Don’t know | ****  ****  ****  **** |
| --- | --- | --- | --- |

*If “No” or “Refused to answer” or “Don’t know” go to question 2.60.*

| 2.58 | For how long before death did ______ have difficulty swallowing? | 1. Months ___ ___  2. Days ___ ___  8. Refused to answer  9. Don’t know | ****  **** |
| --- | --- | --- | --- |
| 2.59 | Was the difficulty with swallowing with solids, liquids, or both? | 1. Solids  2. Liquids  3. Both  8. Refused to answer  9. Don’t know | ****  ****  ****  ****  **** |
| 2.60 | Did ________ have pain upon swallowing? | 1. Yes  2. No  8. Refused to answer  9. Don’t know | ****  ****  ****  **** |

| 2.61 | Did _____ have belly pain? | 1. Yes  2. No  8. Refused to answer  9. Don’t know | ****  ****  ****  **** |
| --- | --- | --- | --- |

*If “No” or “Refused to answer” or “Don’t know” go to question 2.64.*

| 2.62 | For how long before death did ______ have belly pain? | 1. Months ___ ____  2. Days ___ ___  3. Hours ___ ___  8. Refused to answer  9. Don’t know | ****  **** |
| --- | --- | --- | --- |
| 2.63 | Was the pain in the upper or lower belly? | 1. Upper belly  2. Lower belly  8. Refused to answer  9. Don’t know | ****  ****  ****  **** |

| 2.64 | Did _____ belly protrude more than usual? | 1. Yes  2. No  8. Refused to answer  9. Don’t know | ****  ****  ****  **** |
| --- | --- | --- | --- |

*If “No” or “Refused to answer” or “Don’t know” go to question 2.67.*

| 2.65 | For how long before death did ______ have a protruding belly? | 1. Months ___ ___  2. Days ___ ___  8. Refused to answer  9. Don’t know | ****  **** | |
| --- | --- | --- | --- | --- |
| 2.66 | How rapidly did _______ develop the protruding belly? | 1. Rapidly  2. Slowly  8. Refused to answer  9. Don’t know | ****  ****  ****  **** |  |

| 2.67 | Did _____ have any mass in the belly? | 1. Yes  2. No  8. Refused to answer  9. Don’t know | ****  ****  ****  **** |
| --- | --- | --- | --- |

*If “No” or “Refused to answer” or “Don’t know” go to question 2.69.*

| 2.68 | For how long before death did ______ have a mass in the belly? | 1. Months ___ ___  2. Days ___ ___  8. Refused to answer  9. Don’t know | ****  **** |
| --- | --- | --- | --- |

| 2.69 | Did _____ have headaches? | 1. Yes  2. No  8. Refused to answer  9. Don’t know | ****  ****  ****  **** |
| --- | --- | --- | --- |

*If “No” or “Refused to answer” or “Don’t know” go to question 2.72.*

| 2.70 | For how long before death did ______ have headaches? | 1. Hours ___ ___  2. Days ___ ___  8. Refused to answer  9. Don’t know | ****  **** |
| --- | --- | --- | --- |
| 2.71 | Was the onset of the headache fast or slow? | 1. Fast  2. Slow  8. Refused to answer  9. Don’t know | ****  ****  ****  **** |

| 2.72 | Did _____ have a stiff neck? | 1. Yes  2. No  8. Refused to answer  9. Don’t know | ****  ****  ****  **** |
| --- | --- | --- | --- |

*If “No” or “Refused to answer” or “Don’t know” go to question 2.74.*

| 2.73 | For how long before death did ______ have stiff neck? | 1. Months ___ ___  2. Days ___ ___  8. Refused to answer  9. Don’t know | ****  **** |
| --- | --- | --- | --- |

| 2.74 | Did ______ experience a period of loss of consciousness? | 1. Yes  2. No  8. Refused to answer  9. Don’t know | ****  ****  ****  **** |  |  |
| --- | --- | --- | --- | --- | --- |

*If “No” or “Refused to answer” or “Don’t know” go to question 2.78.*

| 2.75 | Did the period of loss of consciousness start suddenly or slowly? | 1. Suddenly  2. Slowly  8. Refused to answer  9. Don’t know | ****  ****  ****  **** |
| --- | --- | --- | --- |
| 2.76 | For how long did the period of loss of consciousness last? | 1. Days ___ ___  2. Months ___ ____  8. Refused to answer  9. Don’t know | ****  **** |
| 2.77 | Did it continue until death? | 1. Yes  2. No  8. Refused to answer  9. Don’t know | ****  ****  ****  **** |

| 2.78 | Did _________ experience a period of confusion at any time in the three months prior to death? | 1. Yes  2. No  8. Refused to answer  9. Don’t know | ****  ****  ****  **** |
| --- | --- | --- | --- |
|  | *If “No” or “Refused to answer” or “Don’t know” go to question 2.81.* | | |
| 2.79 | For how long did the period of confusion last? | 1. Days ___ ___  2. Months ___ ___  8. Refused to answer  9. Don’t know | ****  **** |
| 2.80 | Did the period of confusion start suddenly or slowly? | 1. Suddenly  2. Slowly  8. Refused to answer  9. Don’t know | ****  ****  ****  **** |
| 2.81 | Did ________ experience memory loss at any time in the three months prior to death? | 1. Yes  2. No  8. Refused to answer  9. Don’t know | ****  ****  ****  **** |
| 2.82 | Did _____ have convulsions?  *(Demonstrate)* | 1. Yes  2. No  8. Refused to answer  9. Don’t know | ****  ****  ****  **** |

*If “No” or “Refused to answer” or “Don’t know” go to question 2.85.*

| 2.83 | For how long before death did the convulsions last? | 1. Minutes ___ ___  2. Hours ___ ___  8. Refused to answer  9. Don’t know | ****  **** |
| --- | --- | --- | --- |
| 2.84 | Did the person become unconscious immediately after the convulsions? | 1. Yes  2. No  8. Refused to answer  9. Don’t know | ****  ****  ****  **** |

| 2.85 | Was _______ in any way paralyzed? | 1. Yes  2. No  8. Refused to answer  9. Don’t know | ****  ****  ****  **** |
| --- | --- | --- | --- |

*If “No” or “Refused to answer” or “Don’t know” go to 2.88.*

| 2.86 | For how long before death did _______ have paralysis? | 1. Days ___ ___  2. Months ___ ___  3. Years ___ ___  8. Refused to answer  9. Don’t know | ****  **** |
| --- | --- | --- | --- |
| 2.87 | Which were the limbs or body parts paralyzed?  *Read through the list in sequence and MARK ALL THAT APPLY* | 1. Right side  (arm and leg)  2. Left side  (arm and leg)  3. Lower part of  the body  4. Upper part of  the body  5. One leg only  6. One arm only  7. Whole body  8. Refused to answer  9. Don’t know  10. Other (specify)  _________________ | ****  ****  ****  ****  ****  ****  ****  ****  ****  **** |

| **2.88** | ***STOP.***  ***If the deceased was female then continue to Section 3: Questions for Women.***  ***If the deceased was male then go to Section 4: Alcohol and Tobacco.*** |  |
| --- | --- | --- |

**SECTION 3: QUESTIONS FOR WOMEN**

| 3.1 | Did ________ have any swelling or lump in the breast? | 1. Yes  2. No  8. Refused to answer  9. Don’t know | ****  ****  ****  **** |
| --- | --- | --- | --- |
| 3.2 | Did ________ have any ulcers (pits) in the breast? | 1. Yes  2. No  8. Refused to answer  9. Don’t know | ****  ****  ****  **** |
| 3.3 | Had ________ period’s stopped naturally because of menopause?  *If no, skip to 3.5.* | 1. Yes  2. No  8. Refused to answer  9. Don’t know | ****  ****  ****  **** |
| 3.4 | Did _______ have vaginal bleeding after cessation of menstruation? (post menopausal)  *After asking 3.4, skip to Section 4.* | 1. Yes  2. No  8. Refused to answer  9. Don’t know | ****  ****  ****  **** |
| 3.5 | Did ______ have vaginal bleeding other than her period? (intermenstrual) | 1. Yes  2. No  8. Refused to answer  9. Don’t know | ****  ****  ****  **** |

| 3.6 | Was there excessive vaginal bleeding in the week prior to death? | 1. Yes  2. No  8. Refused to answer  9. Don’t know | ****  ****  ****  **** |
| --- | --- | --- | --- |
| 3.7 | At the time of death was her period overdue? | 1. Yes  2. No  8. Refused to answer  9. Don’t know | ****  ****  ****  **** |

*If “No” or “Refused to answer” or “Don’t know” go to question 3.10.*

| 3.8 | For how many weeks was her period overdue? | 1. Weeks ___ ___  8. Refused to answer  9. Don’t know | ****  **** |
| --- | --- | --- | --- |
| 3.9 | Did she have a sharp pain in the belly shortly before death? | 1. Yes  2. No  8. Refused to answer  9. Don’t know | ****  ****  ****  **** |

| 3.10 | Was she pregnant at the time of death?  *If “No” or “Refused to answer” or “Don’t know”, skip to 3.17.* | 1. Yes  2. No  8. Refused to answer  9. Don’t know | ****  ****  ****  **** |
| --- | --- | --- | --- |
| 3.11 | For how many months was she pregnant? | Months __ ___  8. Refused to answer  9. Don’t know | ****  **** |

| 3.12 | Did ______ die during an abortion? | 1. Yes  2. No  8. Refused to answer  9. Don’t know | ****  ****  ****  **** |
| --- | --- | --- | --- |
|  | *If “Yes” skip to 3.19* | | |
| 3.13 | Did bleeding occur while she was pregnant? | 1. Yes  2. No  8. Refused to answer  9. Don’t know | ****  ****  ****  **** |
|  |  | | |
| 3.14 | Did she have excessive bleeding during labor or delivery? | 1. Yes  2. No  8. Refused to answer  9. Don’t know | ****  ****  ****  **** |
| 3.15 | Did she die during labor or delivery?  *(Labor is the period of time by which contractions are less than 10 minutes apart.)* | 1. Yes  2. No  8. Refused to answer  9. Don’t know | ****  ****  ****  **** |
| 3.16 | For how long was she in labor?  *If she died while in labor, skip to Section 4.* | 1. Hours ___ ____  8. Refused to answer  9. Don’t know | ****  **** |
|  |  | | |
| 3.17 | Did she die within 6 weeks of having an abortion?  *If “Yes”, skip to 3.19.* | 1. Yes  2. No  8. Refused to answer  9. Don’t know | ****  ****  ****  **** |
| 3.18 | Did she die within 6 weeks of childbirth?  If “No” or “Refused to answer” or “Don’t know”, skip to Section 4. | 1. Yes  2. No  8. Refused to answer  9. Don’t know | ****  ****  ****  **** |
| 3.19 | Did she have excessive bleeding after delivery or abortion? | 1. Yes  2. No  8. Refused to answer  9. Don’t know | ****  ****  ****  **** |
| 3.20 | Did ______ have bad smelling vaginal discharge within 6 weeks after delivery or abortion? | 1. Yes  2. No  8. Refused to answer  9. Don’t know | ****  ****  ****  **** |
|  |  |  |  |
|  | ***STOP.***  ***Go to Section 4: Alcohol and Tobacco.*** | | |

**SECTION 4: ALCOHOL AND TOBACCO**

| 4.1 | Did _________ use tobacco? | 1. Yes  2. No  8. Refused to answer  9. Don’t know | ****  ****  ****  **** |
| --- | --- | --- | --- |

*If “No” or “Refused to answer” or “Don’t know” go to question 4.5.*

| 4.2 | What kind of tobacco did _____ use?  *Each site should tailor option four to this question to meet their need* | 1. Cigarettes  2. Pipe  3. Chewing tobacco  4. Local form of tobacco  5. Other (specify)  8. Refused to answer  9. Don’t know | ****  ****  ****  ****    ****  **** |
| --- | --- | --- | --- |

*If “Yes” to chewing tobacco, continue to 4.3. If “Yes” to cigarettes, skip to 4.4. If “No” to chewing tobacco and cigarettes skip to 4.5.*

| 4.3 | How much chewing tobacco did ________ use daily? | 1. Number of “chew” or “dips” per day  ___ ___  8. Refused to answer  9. Don’t know | ****  **** |
| --- | --- | --- | --- |
| 4.4 | How many cigarettes did _____ smoke daily? | 1. Number ___ ___  8. Refused to answer  9. Don’t know | ****  **** |
| 4.5 | Did ______ drink alcohol? | 1. Yes  2. No  8. Refused to answer  9. Don’t know | ****  ****  ****  **** |

*If “No” or “Refused to answer” or “Don’t know” go to question 5.1.*

| 4.6 | Would you say the amount of alcohol _____ drank daily was…? | 1. Low  2. Moderate  3. High  8. Refused to answer  9. Don’t know | ****  ****  ****  ****  **** |
| --- | --- | --- | --- |

**SECTION 5: INJURIES/ACCIDENTS**

| 5.1 | Did ________ suffer from an injury or accident such as a…….?    *Ask respondent each in sequence and mark all to which the respondent indicated “Yes.”* | 1. Road traffic injury  2. Fall  3. Drowning  4. Poisoning  5. Bite or sting by  venomous animal  6. Burn  7. Violence (suicide,  homicide, abuse)  8. No  9. Other injury  (Specify) _________ | ****  ****  ****  ****  ****  ****  ****  ****  **** |
| --- | --- | --- | --- |
|  | *If no boxes are checked, go to Section 6. If at least one box is checked, continue to 5.2.* | | |
| 5.2 | Was the injury or accident self-inflicted? | 1. Yes  2. No  8. Refused to answer  9. Don’t know | ****  ****  ****  **** |

*If “Yes” skip to 5.4.*

|  |  |  |  |
| --- | --- | --- | --- |
| 5.3 | Was the injury or accident intentionally inflicted by someone else? | 1. Yes  2. No  8. Refused to answer  9. Don’t know | ****  ****  ****  **** |

| 5.4 | How long did ________ survive after the injury? | 1. Hours ___ ___  2. Days ___ ____  3. Months ___ ____  4. Years ____ ____  8. Refused to answer  9. Don’t know | ****  **** |
| --- | --- | --- | --- |

**SECTION 6: HEALTH RECORDS**

| 6.1 | Was care sought outside the home while ­­­­­­­­­­­­­­­___________ had this illness? | 1. Yes  2. No  8. Refused to answer  9. Don’t know |        |
| --- | --- | --- | --- |
|  | *If “No” or “Don’t know” or “Refused to answer” go to Question 6.4*. | | |
| 6.2 | Where or from whom did you seek care?  *(CHECK ALL THAT APPLY)* | 1. Traditional Healer 2. Homeopath 3. Religious leader 4. Government Hospital 5. Governmental health center or clinic 6. Private Hospital 7. Community-based practitioner associated with health system 8. Trained birth attendant 9. Private physician 10. Pharmacy, drug seller, store, market 11. Other provider 12. Relative, friend (outside household) 13. Refused to answer   99. Don’t Know |                            |
| 6.3 | *Record the name and address of any hospital, health center or clinic where care was sought:* | |  |
| 6.4 | Do you have any health records that belonged to the deceased? | 1. Yes  2. No  8. Refused to answer  9. Don’t know |        |
|  | *If “No” or “Don’t know” or “Refused to answer” go to question 6.10.* | | |
| 6.5 | Can I see the health records? | 1. Yes  2. No  8. Refused to answer |      |
|  | *If “No” or “Refused to answer” go to question 6.10; If “Yes”, and respondent allows you to see the records, transcribe all the entries* | | |
| 6.6 | *Record the dates of the two most recent visits*  *If not listed, mark 9999* | 1. _ _/_ _/_ _ _ _  dd mm yyyy  2. _ _/_ _/_ _ _ _  dd mm yyyy |  |
| 6.7 | *Record the date of the last note* | *_ _/_ _/_ _ _ _*  dd mm yyyy |  |
| 6.8 | *Transcribe the note:* | | |
|  |
| 6.9 | Was a death certificate issued? | 1. Yes  2. No  8. Refused to answer  9. Don’t know |        |
|  | *If “No” or “Don’t know” or “Refuse to answer” go to Section 7.* | | |
| 6.10 | Can I see the death certificate? | 1. Yes  2. No  8. Refused to answer |      |
|  | *If “No” go to Section 7.* |  |  |
| 6.11 | *Record the immediate cause of death from the certificate.* | ­­­ | |
| 6.12 | *Record the first underlying cause of death from the certificate.* |  | |
| 6.13 | *Record the second underlying cause of death from the certificate.* |  | |
| 6.14 | *Record the third underlying cause of death from the certificate.* |  | |
| 6.15 | *Record the contributing cause(s) of death from the certificate.* |  | |

**SECTION 7: OPEN ENDED RESPONSE AND INTERVIEWER COMMENTS/OBSERVATIONS**

7.1

INSTRUCTIONS TO INTERVIEWER: Say to the respondent: “Thank you for the patient responses to this exhaustive set of questions. Could you please summarize, or tell us in your own words, any additional information about the illness and/or death of your loved one?”

To the Interviewer: Write down what the respondent tells you in his/her own words. Do not prompt except for asking whether there was anything else after the respondent finishes. While recording, underline any unfamiliar terms. You may also use this space to write down your comments and observations about the interview.

|  |
| --- |
|  |
|  |
|  |
|  |
|  |
|  |
|  |
|  |
|  |
|  |
|  |
|  |
|  |
|  |
|  |
|  |

**END OF INTERVIEW**

Thank respondent for their cooperation
