# Supplementary material for: Population Health Metrics Research Consortium gold standard verbal autopsy validation study: design, implementation, and development of analysis datasets
Source: Popul Health Metr. 2011 Aug 4;9:27. doi: 10.1186/1478-7954-9-27 (PMC3160920; doi:10.1186/1478-7954-9-27)
Supplement: Additional file 4 — Child and neonate module of the full verbal autopsy instrument used in the field in the PHMRC study. [file 1478-7954-9-27-S4.DOC]

**POPULATION HEALTH METRICS RESEARCH CONSORTIUM NEONATAL AND CHILD VERBAL AUTOPSY MODULE**

**SECTION 1: BACKGROUND**

| 1.1 | Was the deceased a singleton or multiple birth*? | 1. Singleton 2. Multiple   8. Refuse to answer  9. Don’t know |        |
| --- | --- | --- | --- |
|  | **If two or more children are born at the same time, it is counted as a multiple birth, even if one or more of the babies are born dead. If “Multiple”, ask:* | |  |
|  | *If 1.1 is “Singleton” skip to 1.3* | |  |
| 1.2 | Was this the first, second, or later in the birth order? | 1. First 2. Second 3. Third or more   8. Refuse to answer   1. Don’t know |          |
|  | *If mother is present, go to 1.6. If mother is not present at the interview, ask:* | |  |
| 1.3 | Is the mother still alive? | 1. Yes 2. No |    |
|  | *If “Yes”, go to 1.6.* |  |  |
| 1.4 | Did the mother die during or after the delivery? | 1. During 2. After   8. Refused to answer   1. Don’t know |        |
|  | *If “During” delivery, go to 1.6.* |  |  |
| 1.5 | How long after the delivery did the mother die?  *(Less than 24 hours = 00 days. Use 1 month = 28 days to determine the number of months.)* | 1. __ __ days OR 2. __ __ months   8. Refused to answer  9. Don’t know |    |
|  |  |  |  |
| 1.6 | Where was the deceased born? | 1. Hospital 2. Other health facility 3. On route to hospital or other health facility 4. Home 5. Other _________   8. Refused to answer  9. Don’t know |                |
| 1.7 | At the time of the delivery was the deceased:  *Read the question and slowly read the first 4 choices. Respondent should hear all four choices and then respond.*  *(Show photos)* | 1. Very small  2. Smaller than usual  3. About average  4. Larger than usual  8. Refused to answer  9. Don’t know |            |
| 1.8 | What was the weight of the deceased at birth? | 1. __ __ __ __ pounds  8. Refused to answer  9. Don’t know |    |
| 1.9 | What was the sex of the deceased? | 1. Male 2. Female   8. Refused to answer  9. Don’t know |        |
| 1.10 | What was the delivery date? | 1. *_ _/_ _/_ _ _ _*  dd/mm/yyyy  8. Refused to answer  9. Don’t know |    |
| 1.11 | Was the child born alive or dead? | 1. Alive   2. Dead  8. Refused to answer  9. Don’t know |        |
| 1.12 | Did the baby ever cry? | 1. Yes 2. No   8. Refused to answer  9. Don’t know |        |
| 1.13 | Did the baby ever move? | 1. Yes 2. No   8. Refused to answer  9. Don’t know |        |
| 1.14 | Did the baby ever breathe? | 1. Yes 2. No   8. Refused to answer  9. Don’t know |        |
| 1.15 | *INTERVIEWER ONLY: Refer to questions 1.12, 1.13, and 1.14. If all three responses are “No” then check “Yes” below. Otherwise, check “No.”*  *Yes No* | |  |
|  | ***STOP.***  ***If you answered “Yes” to 1.15 (stillbirth), then go to 1.16.***  ***If you answered “No” to 1.15 (live birth), go to 1.20.*** | |  |

| 1.16 | Were there any bruises or signs of injury on the baby’s body at birth? | 1. Yes 2. No   8. Refused to answer   1. Don’t know |        |  |
| --- | --- | --- | --- | --- |
| 1.17 | Was the baby’s body (skin and tissue) pulpy? | 1. Yes 2. No   8. Refused to answer   1. Don’t know |        |  |
| 1.18 | Was any part of the baby physically abnormal at time of delivery? (for example: body part too large or too small, additional growth on body) | 1. Yes 2. No   8. Refused to answer  9. Don’t know |        |  |
|  | *If “No” or “Don’t know” or “Refused to answer” go to Section 2.* | |  | |
| 1.19 | What were the abnormalities?  *MARK ALL THAT APPLY (Show photos)* | 1. Head size very small at time of birth?    2. Head size very large at time of birth?    3. Mass defect on the back of head or spine    4. Other  Specify________  8. Refused to answer |          |  |
|  | ***STOP.***  ***After completing 1.19, continue to Section 2: MATERNAL HISTORY.*** | |  | |

| 1.20 | How old was the baby/child when the fatal illness started?  *(Less than 24 hours = 00 days. Use 1 month = 28 days to determine the number of months.)* | 1. __ __ days (if less than one month)  2. __ __ months (if less than one year)  3. ___ ___ years (one year or older)  8. Refused to answer  9. Don’t know |    |
| --- | --- | --- | --- |
| 1.21 | How long did the illness last?  *(Less than 24 hours = 00 days. Use 1 month = 28 days to determine the number of months.)* | 1. __ __ days OR  2. __ __ months  8. Refused to answer  9. Don’t know |    |
| 1.22 | Where did the deceased die? | 1. Hospital  2. Other health facility  3. On route to hospital or health facility  4. Home  5. Other (specify________)  8. Refused to answer  9. Don’t know |              |
| 1.23 | *For deaths at a hospital or health facility, record facility name and address:* | |  |
|  |  | |  |
| 1.24 | What was the date of death? | 1. *_ _/_ _/_ _ _ _*  dd/mm/yyyy  8. Refused to answer  9. Don’t know |    |
| 1.25 | How old was the deceased at the time of death? | 1. __ __ days (if less than one month)  2. __ __ months (if less than one year)  3. ___ ___ years (one year or older)  8. Refused to answer  9. Don’t know |    |
|  | *(Use one month = 28 days to determine the number of months)* | |  |
| 1.26 | *Mark the baby’s age at the time of death.* | 1. Less than 28   days old   1. 28 days—11 years |    |
|  | *INTERVIEWER: If 1.25 is “Refused to answer” or “Don’t know” use your best judgment to answer 1.26.* | | |
| **STOP.**  **If the child is less than 28 days old, continue to SECTION 2: MATERNAL HISTORY.**  **If the child is 28 days—11 years old, go to SECTION 4: INFANT AND CHILD DEATHS** | | | |

**SECTION 2: MATERNAL HISTORY**

| 2.1 | Was the late part of the pregnancy (defined as the last 3 months), labor, or delivery complicated by any of the following problems? | 1. You *(the mother*) had convulsions 2. You *(the mother)* had high blood pressure 3. You *(the mother)* had severe anemia 4. You *(the mother)* had diabetes   5. Child delivered not head first  6. Cord delivered first  7. Cord around child’s neck  8. Excessive bleeding   1. Fever during labor   10. No complications  11. Refused to answer  12. Don’t know |                        | | |
| --- | --- | --- | --- | --- | --- |
| *(Read each complication and mark all that apply.)*  *(Read “the mother” if the mother is not the respondent.)* | |
| 2.2 | How many months long was the pregnancy? | 1. __ __ months  8. Refused to answer  9. Don’t know |    | | |
|  | *If number of months is known go to 2.4.* | |  | | |
| 2.3 | Did the pregnancy end early, on time, or late? | 1. Early 2. On time 3. Late   8. Refused to answer  9. Don’t know  D |          | | |
| 2.4 | Was the baby moving in the last few days before the birth? | 1. Yes 2. No   8. Refused to answer   1. Don’t know |        | | |
| 2.5 | When did you *(the mother)* last feel the baby move?  *(Read “the mother” if the mother is not the respondent.)* | 1. __ __ hours before delivery   1. OR   2. __ __ days before delivery  8. Refused to answer  9. Don’t know |    | | |
| 2.6 | Did the water break before labor or during labor?  (*Note: labor begins when contractions are no more than 10 minutes apart.*) | 1. Before 2. During   8. Refused to answer  9. Don’t know |          | | |
|  | *If “During” delivery, go to 2.8.* | |  | | |
| 2.7 | How much time before labor did the water break? | 1. Less than one day  2. One day or more  8. Refused to answer  9. Don’t know |        | | |
| 2.8 | What was the color of the liquor when the water broke? | 1. Green or brown 2. Clear (normal) 3. Other (specify_______)   8. Refused to answer  9. Don’t know |          | | |
| 2.9 | Was the liquor foul smelling? | 1. Yes 2. No   8. Refused to answer   1. Don’t know |        | | |
| 2.10 | How much time did the labor and delivery take?  *(Less than 1 hour= “00”)* | 1. ___ ___ hours  8. Refused to answer  9. Don’t know | |    | |
| 2.11 | Did you *(the mother)* receive any vaccinations since reaching adulthood including during this pregnancy?  *(Read “the mother” if the mother is not the respondent.)* | 1. Yes 2. No   8. Refused to answer   1. Don’t know | |        | |
|  | *If “No” or “Don’t know” or “Refused to answer” go to question 2.13.* | | |  | |
| 2.12 | How many doses? | 1. One 2. Two 3. Three 4. Four 5. Five or more   8. Refused to answer  9. Don’t know | |              | |
| 2.13 | Where did the delivery occur? | 1. Hospital 2. Other health facility 3. On route to hospital   or other health facility   1. Home 2. Other   Specify_____­____ 8. Refused to answer  9. Don’t know | |              | |
| 2.14 | *For deliveries at hospital or other health facility, record facility name and address:* | | | | |
| 2.15 | Who delivered the baby?  *(Read “the mother” if the mother is not the respondent.)* | 1. Doctor 2. Nurse/midwife 3. Relative 4. Self *(the mother)* 5. Traditional birth attendant 6. Other(specify_____)   8. Refused to answer  9. Don’t know | |                | |
| 2.16 | *If nurse/midwife in the community, record her name and address:* | | |  | |
|  |  | | | | |
|  |
|  |
| 2.17 | Was the delivery…?  *(Read the choices and mark ONE.)* | 1. Vaginal with forceps  2. Vaginal w/out forceps  3. Vaginal don’t know  4. C-Section  8. Refused to answer  9. Don’t know | | |            |
| 2.18 | During labor but before delivery, did you *(the mother)* receive any kind of injection?  *(Read “the mother” if the mother is not the respondent.)* | 1. Yes 2. No   8. Refused to answer   1. Don’t know | | |        |
|  | **STOP.**  **Refer back to question 1.15. If you answered “Yes,” go to question 5.4 (*Section 5: Health Records)***  **If you answered “No,” continue to Section 3: Neonatal Deaths.** | | | |  |

**SECTION 3: NEONATAL DEATHS**

| 3.1 | Were there any bruises or signs of injury on the baby’s body at birth? | 1. Yes 2. No   8. Refused to answer   1. Don’t know |        |
| --- | --- | --- | --- |
| 3.2 | Was any part of the baby physically abnormal at time of delivery? (for example: body part too large or too small, additional growth on body) | 1. Yes 2. No   8. Refused to answer   1. Don’t know |        |
|  | *If “No” or “Don’t know” or “Refused to answer” go to 3.4.* | | |
| 3.3 | What were the abnormalities?  *MARK ALL THAT APPLY*  *(Show photos)* | 1. Head size very small at time of birth?  2. Head size very large at time of birth?    3. Mass defect on the back of head or spine    4. Other  (Specify: ___________________)  8. Refused to answer |          |
| 3.4 | Did the baby breathe immediately after birth? | 1. Yes 2. No   8. Refused to answer  9. Don’t know |        |
|  | *If “No” go to 3.6.* |  |  |
| 3.5 | Did the baby have difficulty breathing? | 1. Yes 2. No   8. Refused to answer  9. Don’t know |        |
| 3.6 | Was anything done to try to help the baby breathe at birth? | 1. Yes  2. No  8. Refused to answer  9. Don’t know |        |
| 3.7 | Did the baby cry immediately after birth? | 1. Yes  2. No  8. Refused to answer  9. Don’t know |        |
|  | *If “Yes” go to 3.9.* | |  |
| 3.8 | How long after birth did the baby first cry?  *(MARK ONE)*  *If “Never” go to 3.11.* | 1. Within 5 minutes  2. Within 6-30 minutes  3. More than 30 minutes  4. Never  8. Refused to answer  9. Don’t know |            |
| 3.9 | Did the baby stop being able to cry? | 1. Yes  2. No  8. Refused to answer  9. Don’t know |        |
|  | *If “No” or “Don’t know” or “Refused to answer” go to question 3.11.* | | |
| 3.10 | How long before the baby died did the baby stop crying? | 1. Less than one day 2. One day or more   8. Refused to answer  9. Don’t know |        |
| 3.11 | Was the baby able to suckle in a normal way during the first day of life? | 1. Yes  2. No  8. Refused to answer  9. Don’t know |        |
|  | *If “Yes” go to 3.13.* |  |  |
| 3.12 | Did the baby ever suckle in a normal way? | 1. Yes  2. No  8. Refused to answer  9. Don’t know |        |
|  | *If “No” or “Don’t know” or “Refused to answer” go to 3.17.* | | |
| 3.13 | Did the baby stop being able to suckle in a normal way? | 1. Yes  2. No  8. Refused to answer  9. Don’t know |        |
|  | *If “No” or “Don’t know” or “Refused to answer” go to 3.17.* | | |
| 3.14 | How long after birth did the baby stop suckling?  *(Less than 1 day= “00”)* | Record actual days   1. ___ ___   8. Refused to answer  9. Don’t know |    |
| 3.15 | How long before he/she died did the baby stop suckling? | 1. Less than one day  2. One day or more  8. Refused to answer  9. Don’t know |        |
| 3.16 | Was the baby able to open his/her mouth at the time he/she stopped sucking? | 1. Yes  2. No  8. Refused to answer  9. Don’t know |        |
| 3.17 | During the illness that led to death, did the baby have difficult breathing? | 1. Yes  2. No  8. Refused to answer  9. Don’t know |        |
|  | *If “No” or “Don’t know” or “Refused to answer” go to 3.20.* | | |
| 3.18 | At what age did the difficult breathing start?    *(Less than 1 day= “00”)* | 1. ___ ___ days   8. Refused to answer  9. Don’t know |    |
| 3.19 | For how many days did the difficult breathing last?  *(Less than 1 day= “00”)* | 1. ___ ___ days   8. Refused to answer  9. Don’t know |    |
| 3.20 | During the illness that led to death, did the baby have fast breathing? | 1. Yes  2. No  8. Refused to answer  9. Don’t know |        |
|  | *If “No” or “Don’t know” or “Refused to answer” go to 3.23.* | | |
| 3.21 | At what age did the fast breathing start?  *(Less than 1 day= “00”)* | 1. ___ ___ days   8. Refused to answer  9. Don’t know |    |
| 3.22 | For how many days did the fast breathing last?  *(Less than 1 day= “00”)* | 1. ___ ___ days  8. Refused to answer  9. Don’t know |    |
| 3.23 | During the illness that led to death, did the baby have indrawing of the chest?    *(Show photo)* | 1. Yes  2. No  8. Refused to answer  9. Don’t know |        |
| 3.24 | During the illness that led to death, did the baby have grunting?  (*Demonstrate*) | 1. Yes  2. No  8. Refused to answer  9. Don’t know |        |
| 3.25 | During the illness that led to death did the baby have spasms or convulsions? | 1. Yes  2. No  8. Refused to answer  9. Don’t know |        |
| 3.26 | During the illness that led to death, did the baby have fever? | 1. Yes  2. No  8. Refused to answer  9. Don’t know |        |
|  | *If “No” or “Don’t know” or “Refused to answer” go to 3.29.* | | |
| 3.27 | At what age did the fever start?  *(Less than 1 day= “00”)* | 1. ___ ___ days  8. Refused to answer  9. Don’t know |    |
| 3.28 | How many days did the fever last?  *(Less than 1 day= “00”)* | 1. ___ ___ days  8. Refused to answer  9. Don’t know |    |
| 3.29 | During the illness that led to death, did the baby become cold to touch? | 1. Yes  2. No  8. Refused to answer  9. Don’t know |        |
|  | *If “No” or “Don’t know” or “Refused to answer” go to 3.32.* | | |
| 3.30 | At what age did the baby start feeling cold to touch?  *(Less than 1 day= “00”)* | 1. ___ ___ days   8. Refused to answer  9. Don’t know |    |
| 3.31 | How many days did the baby feel cold to touch?  *(Less than 1 day= “00”)* | 1. ___ ___ days   8. Refused to answer  9. Don’t know |    |
| 3.32 | During the illness that led to death, did the baby become lethargic, after a period of normal activity? | 1. Yes  2. No  8. Refused to answer  9. Don’t know |        |
| 3.33 | During the illness that led to death, did the baby become unresponsive or unconscious? | 1. Yes  2. No  8. Refused to answer  9. Don’t know |        |
| 3.34 | During the illness that led to death, did the baby have a bulging fontanelle?    *(Show photo)* | 1. Yes  2. No  8. Refused to answer  9. Don’t know |        |
| 3.35 | During the illness that led to death, did the baby have pus drainage from the umbilical cord stump? | 1. Yes  2. No  8. Refused to answer  9. Don’t know |        |
| 3.36 | During the illness that led to death, did the baby have redness of the umbilical cord stump? | 1. Yes  2. No  8. Refused to answer  9. Don’t know |        |
|  | *If “No” or “Don’t know” or “Refuse to answer” go to 3.38.* | | |
| 3.37 | Did the redness of the umbilical cord stump extend onto the abdominal skin? | 1. Yes  2. No  8. Refused to answer  9. Don’t know |        |
| 3.38 | During the illness that led to death, did the baby have skin bumps containing pus or a single large area with pus? | 1. Yes  2. No  8. Refused to answer  9. Don’t know |        |
| 3.39 | During the illness that led to death, did the baby have ulcer(s) (pits)? | 1. Yes  2. No  8. Refused to answer  9. Don’t know |        |
| 3.40 | During the illness that led to death, did the baby have an area(s) of skin with redness and swelling? | 1. Yes  2. No  8. Refused to answer  9. Don’t know |        |
| 3.41 | During the illness that led to death, did he/she have areas of the skin that turned black? | 1. Yes 2. No   8. Refused to answer   1. Don’t know |        |
| 3.42 | During the illness that led to death, did the baby bleed from anywhere? | 1. Yes  2. No  8. Refused to answer  9. Don’t know |        |
|  | *If “No” or “Don’t know” or “Refused to answer” go to 3.44.* | | |
| 3.43 | *Record from where did the baby bleed:* | |  |
| 3.44 | During the illness that led to death, did he/she have more frequent loose or liquid stools than usual? | 1. Yes  2. No  8. Refused to answer  9. Don’t know |        |
|  | *If “No” or “Don’t know” or “Refused to answer” go to 3.46.* | | |
| 3.45 | How many stools did the baby have on the day that diarrhea/loose liquid stools were most frequent? | 1. ___ ___ stools  8. Refused to answer  9. Don’t know |    |
| 3.46 | During the illness that led to death, did he/she vomit everything? | 1. Yes  2. No  8. Refused to answer  9. Don’t know |        |
| 3.47 | During the illness that led to death, did he/she have yellow skin? | 1. Yes  2. No  8. Refused to answer  9. Don’t know |        |
| 3.48 | During the illness that led to death, did the baby have yellow eyes? | 1. Yes  2. No  8. Refused to answer  9. Don’t know |        |
| 3.49 | Did the infant appear to be healthy and then just die suddenly? | 1. Yes  2. No  8. Refused to answer  9. Don’t know |        |

| **STOP.**  **END OF NEONATAL DEATHS SECTION**  **GO TO SECTION 5: HEALTH RECORDS SECTION** |
| --- |

**SECTION 4: INFANT AND CHILD DEATHS**

| 4.1 | During the illness that led to death, did ____________ have a fever? | 1. Yes  2. No  8. Refused to answer  9. Don’t know |        |
| --- | --- | --- | --- |
|  | *If “No” or “Don’t know” or “Refused to answer” go to 4.6.* | | |
| 4.2 | How many days did the fever last? | 1. Less than 24 hours  2. __ __ days  8. Refused to answer  9. Don’t know |      |
| 4.3 | Did the fever continue until death? | 1. Yes  2. No  8. Refused to answer  9. Don’t know |        |
|  | *If “No” or “Don’t know” or “Refused to answer” go to 4.6.* | | |
| 4.4 | How severe was the fever? | 1. Mild  2. Moderate  3. Severe  8. Refused to answer  9. Don’t know |          |
| 4.5 | What was the pattern of fever? | 1. Continuous  2. On and off  3. Only at night  8. Refused to answer  9. Don’t know |          |
| 4.6 | During the illness that led to death, did _____________ have more frequent loose or liquid stools than usual? | 1. Yes  2. No  8. Refused to answer  9. Don’t know |        |
|  | *If “No” or “Don’t know” or “Refused to answer” go to 4.12.* | | |
| 4.7 | How many stools did __________ have on the day that loose liquid stools were most frequent? | 1. ___ ___ stools  8. Refused to answer  9. Don’t know |    |
| 4.8 | How many days before death did the frequent loose or liquid stools start? | 1. Less than 24 hrs  2. __ __ days  8. Refused to answer  9. Don’t know |        |
|  | *If Less than 24 hrs, go to 4.12.* |  |  |
| 4.9 | Did the frequent loose or liquid stools continue until death? | 1. Yes  2. No  8. Refused to answer  9. Don’t know |        |
|  | *If “Yes” go to 4.11.* |  |  |
| 4.10 | How many days before death did the loose or liquid stools stop? | 1. Less than 24 hrs  2. __ __ days  8. Refused to answer  9. Don’t know |      |
| 4.11 | Was there visible blood in the loose or liquid stools? | 1. Yes  2. No  8. Refused to answer  9. Don’t know |        |
| 4.12 | During the illness that led to death, did the child have a cough? | 1. Yes  2. No  8. Refused to answer  9. Don’t know |        |
|  | *If “No” or “Don’t know” or “Refused to answer” go to 4.16.* | | |
| 4.13 | For how many days did the cough last? | 1. __ __days  8. Refused to answer  9. Don’t know |    |
| 4.14 | Was the cough very severe? | 1. Yes  2. No  8. Refused to answer  9. Don’t know |        |
| 4.15 | Did the child vomit after he/she coughed? | 1. Yes  2. No  8. Refused to answer  9. Don’t know |        |
| 4.16 | During the illness that led to death, did _____________ have difficult breathing? | 1. Yes  2. No  8. Refused to answer  9. Don’t know |        |
|  | *If “No” or “Don’t know” or “Refused to answer” go to 4.18.* | | |
| 4.17 | For how many days did the difficult breathing last? | 1. __ __ days  8. Refused to answer  9. Don’t know |    |
| 4.18 | During the illness that led to death, did _____________ have fast breathing? | 1. Yes  2. No  8. Refused to answer  9. Don’t know |        |
|  | *If “No” or “Don’t know” or “Refused to answer” go to 4.20.* | | |
| 4.19 | For how many days did the fast breathing last? | 1. __ __ days  8. Refused to answer  9. Don’t know |    |
|  | ***Note to Interviewer: If BOTH 4.16 and 4.18 are “No” go to 4.25.*** | | |
| 4.20 | During the illness that led to death, did he/she have indrawing of the chest? | 1. Yes  2. No  8. Refused to answer  9. Don’t know |        |
| 4.21 | During the illness that led to death, did his/her breathing sound like any of the following:  (*Demonstrate each sound*) |  |  |
| 4.22 | Stridor | 1. Yes  2. No  8. Refused to answer  9. Don’t know |        |
| 4.23 | Grunting | 1. Yes  2. No  8. Refused to answer  9. Don’t know |        |
| 4.24 | Wheezing | 1. Yes  2. No  8. Refused to answer  9. Don’t know |        |
| 4.25 | Did ____________ experience any generalized convulsions or fits during the illness that led to death? | 1. Yes  2. No  8. Refused to answer  9. Don’t know |        |
| 4.26 | Was _____________ unconscious during the illness that led to death? | 1. Yes  2. No  8. Refused to answer  9. Don’t know |        |
|  | *If “No” or “Don’t know” or “Refused to answer” go to 4.28* | | |
| 4.27 | How long before death did unconsciousness start? | 1. Less than 6 hours  2. 6-23 hours  3. 24 hours or more  8. Refused to answer  9. Don’t know |          |
| 4.28 | Did ____________ have a stiff neck during the illness that led to death?  (*Demonstrate*) | 1. Yes  2. No  8. Refused to answer  9. Don’t know |        |
| 4.29 | Did ____________ have a bulging fontanelle during the illness that led to death?  *(Show photo)* | 1. Yes  2. No  8. Refused to answer  9. Don’t know |        |
| 4.30 | During the month before he/she died, did _____________ have a skin rash? | 1. Yes  2. No  8. Refused to answer  9. Don’t know |        |
|  | *If “No” or “Don’t know” or “Refused to answer” go to 4.35.* | | |
| 4.31 | Where was the rash? | 1. Face  2. Trunk/Abdomen  3. Extremities  4. Everywhere  8. Refused to answer  9. Don’t know |            |
| 4.32 | Where did the rash start? | 1. Face  2. Trunk/Abdomen  3. Extremities  4. Everywhere  8. Refused to answer  9. Don’t know |            |
| 4.33 | How many days did the rash last? | 1. __ __ days  8. Refused to answer  9. Don’t know |    |
| 4.34 | Did the rash have blisters containing clear fluid? | 1. Yes  2. No  8. Refused to answer  9. Don’t know |        |
| 4.35 | During the illness that led to death, did __________’s limbs (legs, arms) become very thin?  *(Show photo)* | 1. Yes  2. No  8. Refused to answer  9. Don’t know |        |
| 4.36 | During the illness that led to death, did __________ have swollen legs or feet? | 1. Yes  2. No  8. Refused to answer  9. Don’t know |        |
|  | *If “No” or “Don’t know” or “Refused to answer” go to 4.38.* | | |
| 4.37 | How long did the swelling last? | 1. __ __ days OR  2. __ __ weeks  8. Refused to answer  9. Don’t know |    |
| 4.38 | During the illness that led to death, did ____________’s skin flake off in patches? | 1. Yes  2. No  8. Refused to answer  9. Don’t know |        |
| 4.39 | Did ____________’s hair change in color to a reddish or yellowish color? | 1. Yes  2. No  8. Refused to answer  9. Don’t know |        |
| 4.40 | Did ____________ have a protruding belly? | 1. Yes  2. No  8. Refused to answer  9. Don’t know |        |
| 4.41 | During the illness that led to death, did __________ suffer from “lack of blood” or “pallor”? | 1. Yes  2. No  8. Refused to answer  9. Don’t know |        |
| 4.42 | During the illness that led to death, did ____________ have swelling in the armpits? | 1. Yes  2. No  8. Refused to answer  9. Don’t know |        |
| 4.43 | During the illness that led to death, did ____________ have a whitish rash inside the mouth or on the tongue? | 1. Yes  2. No  8. Refused to answer  9. Don’t know |        |
| 4.44 | During the illness that led to death, did ­________ bleed from anywhere? | 1. Yes  2. No  8. Refused to answer  9. Don’t know |        |
|  | *If “No” or “Don’t know” or “Refused to answer” go to 4.46.* | | |
| 4.45 | *Record from where ­­­he/she bled:* | |  |
| 4.46 | During the illness that led to death, did he/she have areas of the skin that turned black? | 1. Yes  2. No  8. Refused to answer  9. Don’t know |        |
| 4.47 | Did ______ suffer an injury or accident such as…?  *Ask respondent each in sequence and mark all to which the respondent indicated “Yes.”* | 1. Road traffic crash/ injury  2. Fall  3. Drowning  4. Poisoning  5. Bite or sting by venomous animals  6. Fire  7. Violence (suicide, homicide, abuse)  8. Other injury  ___________________  9. Refused to answer  10. Don’t know |                    |
|  | *If no boxes are marked, go to section 5. If at least one box is marked, continue to Question 4.48.* | | |
| 4.48 | Was the injury or accident intentionally inflicted by someone else? | 1. Yes  2. No  8. Refused to answer  9. Don’t know |        |
| 4.49 | How long did ____________ survive after the injury or accident?  *Less than 1 hour=00.* | 1. Hours ___ ___  2. Days ___ ___  8. Refused to answer  9. Don’t know |    |
|  |  |  |  |
|  | **STOP.**  **END OF INFANT AND CHILD DEATHS SECTION**  **GO TO SECTION 5: HEALTH RECORDS SECTION** | |  |

**SECTION 5: HEALTH RECORDS**

| 5.1 | Was care sought outside the home while the deceased had this illness? | 1. Yes  2. No  8. Refused to answer  9. Don’t know |        |
| --- | --- | --- | --- |
|  | *If “No” or “Don’t know” or “Refused to answer” go to Question 5.10*. | | |
| 5.2 | Where or from whom did you seek care?  *(CHECK ALL THAT APPLY)* | 1. Traditional Healer 2. Homeopath 3. Religious leader 4. Government Hospital 5. Governmental health center or clinic 6. Private Hospital 7. Community-based practitioner associated with health system 8. Trained birth attendant 9. Private physician 10. Pharmacy, drug seller, store, market 11. Other provider 12. Relative, friend (outside household) 13. Refused to answer   99. Don’t Know |                        |
| 5.3 | *Record the name and address of any hospital, health center or clinic where care was sought:* | |  |
| 5.4 | Do you have any health records that belonged to the deceased? | 1. Yes  2. No  8. Refused to answer  9. Don’t know |        |
|  | *If “No” or “Don’t know” or “Refused to answer” go to question 5.10.* | | |
| 5.5 | Can I see the health records? | 1. Yes  2. No  8. Refused to answer |      |
|  | *If “No” or “Refused to answer” go to question 5.10; If “Yes”, and respondent allows you to see the records, transcribe all the entries* | | |
| 5.6 | *Record the dates of the two most recent visits*  *If not listed, mark 9999* | 1. _ _/_ _/_ _ _ _  dd mm yyyy  2. _ _/_ _/_ _ _ _  dd mm yyyy |  |
| 5.7 | *Record the two most recent weights on those dates*  *If don’t know, mark 9999* | 1. grams _ _ _ _  2. grams _ _ _ _ |  |
| 5.8 | *Record the date of the last note.* | _ _/_ _/_ _ _ _  dd mm yyyy |  |

| 5.9 | *Transcribe the note:* | | |
| --- | --- | --- | --- |
|  |
| 5.10 | Was a death certificate issued? | 1. Yes  2. No  8. Refused to answer  9. Don’t know |        |
|  | *If “No” or “Don’t know” or “Refuse to answer” go to question 5.17.* | | |
| 5.11 | Can I see the death certificate? | 1. Yes  2. No  8. Refused to answer |      |
|  | *If “No” go to question 5.17.* |  |  |
| 5.12 | *Record the immediate cause of death from the certificate.* | ­­­ | |
| 5.13 | *Record the first underlying cause of death from the certificate.* |  | |
| 5.14 | *Record the second underlying cause of death from*  *the certificate.* |  | |
| 5.15 | *Record the third underlying cause of death from*  *the certificate.* |  | |
| 5.16 | *Record the contributing cause(s) of death from the certificate.* |  | |
| 5.17 | Has the deceased’s (biological) mother ever been tested for “HIV”? | 1. Yes  2. No  8. Refused to answer  9. Don’t know |        |
|  | *If “No” or “Don’t know” or “Refuse to answer” go to question 5.19.* | | |
| 5.18 | Was the “HIV” test ever positive? | 1. Yes  2. No  8. Refused to answer  9. Don’t know |        |
| 5.19 | Has the deceased’s (biological) mother ever been told she had “AIDS” by a health worker? | 1. Yes  2. No  8. Refused to answer  9. Don’t know |        |

**SECTION 6: OPEN ENDED RESPONSE AND INTERVIEWER COMMENTS/OBSERVATIONS**

**INSTRUCTIONS TO INTERVIEWER:** Ask the respondent: “Thank you for the patient responses to this exhaustive set of questions. Could you please summarize, or tell us in your own words, any additional information about the illness and/or death of your loved one?”

To the Interviewer: Write down what the respondent tells you in his/her own words. Do not prompt except for asking whether there was anything else after the respondent finishes. While recording, underline any unfamiliar terms. You may also use this space to write down your comments and observations about the interview.

___________________________________________________________________________________________________________________________________________________________________________________________________________________________________________________________________________________________________________________________________________________________________________________________________

___________________________________________________________________________________________________________________________________________________________________________________________________________________________________________________________________________________________________________________________________________________________________________________________________

___________________________________________________________________________________________________________________________________________________________________________________________________________________________________________________________________________________________________________________________________________________________________________________________________

___________________________________________________________________________________________________________________________________________________________________________________________________________________________________________________________________________________________________________________________________________________________________________________________________

___________________________________________________________________________________________________________________________________________________________________________________________________________________________________________________________________________________________________________________________________________________________________________________________________

___________________________________________________________________________________________________________________________________________________________________________________________________________________________________________________________________________________________________________________________________________________________________________________________________

___________________________________________________________________________________________________________________________________________________________________________________________________________________________________________________________________________________________________________________________________________________________________________________________________

___________________________________________________________________________________________________________________________________________________________________________________________________________________________________________________________________________________________________________________________________________________________________________________________________

____________________________________________________________________________________________________________________________________________________________________________________________________________________________________________________________________________________________________________________________________________________________________________________________________________________________________________________________________________________________________________________________________

**END OF INTERVIEW.**

**THANK RESPONDENT FOR PARTICIPATION**
