# Supplementary material for: Population Health Metrics Research Consortium gold standard verbal autopsy validation study: design, implementation, and development of analysis datasets
Source: Popul Health Metr. 2011 Aug 4;9:27. doi: 10.1186/1478-7954-9-27 (PMC3160920; doi:10.1186/1478-7954-9-27)
Supplement: Additional file 6 — Gold standard (GS) definitions in the PHMRC study for adults. [file 1478-7954-9-27-S6.DOC]

**Adult Gold Standard Diagnoses**

Level 1 = Diagnosis of a particular condition with the highest level of certainty possible for that condition, consisting of either an appropriate laboratory test or x-ray with positive findings and/or medically observed and documented appropriate illness sign(s).

Level 2A = Diagnosis of a particular condition with a high level of certainty, consisting of medically observed and documented appropriate illness or sign(s).

Level 2B= Diagnosis of a particular condition with reasonable certainty but not meeting Level 1 or Level 2A criteria; this category was developed especially for cancer and HIV diagnoses where records are not available.

Level 3 = Cases which would be considered for a gold standard diagnosis but do not meet gold standard criteria: “possible gold standard cases.” This level is designed to exclude possible gold standard cases from the residual categories; no VAs should be collected for any level 3B causes of death.

**Notes:**

1. To be acceptable, illness signs must be observed and documented in a medical record by a physician or clinical officer, unless otherwise specifically noted.

2. Level 1 diagnosis should be the standard used for all gold standard cases. Only if it proves impossible to gather enough cases of a particular condition is it allowable to use the Level 2 diagnosis. For all causes, an autopsy report is acceptable as a gold standard confirmation. (Details are provided below.)

3. Residual Categories: In addition to the priority gold standard causes listed below, the data analysis method requires us to collect a sample of deaths from non-priority causes. These deaths will be grouped into residual categories. Thus, the residual categories will include deaths that occur from non-priority causes, clustered according to Global Burden of Disease cause spans to allow for a balanced distribution of residual causes in the data. The purpose of the Level 3 diagnosis is to prevent possible gold standard deaths from inclusion in the residual categories. If there is a suspicion that the death may be due to one of the priority gold standards, the death does not qualify for the residual category and should be excluded.

4. Several diagnoses are commonly associated with gold standard causes of deaths (co-morbid conditions) or represent a terminal process important for diagnosis. Criteria for co-morbid conditions are included in Appendix A. Criteria for terminal conditions are included in Appendix B.

**I. Adult Infectious Diseases**

**AIDS (A)**

Level 1 Positive for HIV on ELISA test, or Western Blot, or positive for two HIV rapid tests (based on the local standard of care)

PLUS one of the following:

- Extrapulmonary cryptococcosis including meningitis
- Disseminated non-tuberculosis mycobacteria infection
- Progressive multifocal leucoencephalopathy
- Candida of trachea, bronchi or lungs
- Cryptosporidiosis
- Isosporiasis
- Visceral herpes simplex infection
- Cytomegalovirus infection (retinitis of an organ other than liver, spleen or lymph node)
- Any disseminated mycosis (e.g. histoplasmosis, coccidiomycosis)
- Recurrent non-typhoidal salmonella septicaemia
- Lymphoma (cerebral or B-cell non-Hodgkin)
- Invasive cervical carcinoma
- HIV wasting syndrome
- Pneumocystis pneumonia
- Chronic herpes simplex infection (orolabial, genital or anorectal of more than one month’s duration)
- Esophageal candidiasis
- Kaposi’s sarcoma
- CNS toxoplasmosis

Level 2B Patient receiving treatment with ARV where the basis for the initial diagnosis is no longer available

Level 3 Clinical evidence of AIDS in the absence of HIV testing

AIDS smear positive for AFB

**AIDS with TB (A)**

Level 1 Both of the following:

- Positive for HIV on ELISA test, or Western Blot, or positive for two HIV rapid tests (based on the local standard of care)
- Culture positive for M.tuberculosis

Level 3 Evidence suggesting both TB and AIDS, including smear positive, but does not meet Level 1 criteria above

**Diarrhea (A)**

Level 1 Both of the following:

- Reported liquid or watery or loose stools 3+ times a day for at least 1 day
- Observed dehydration
- Observed liquid or semi-liquid or watery stools

Level 3 Death associated with diarrhea not meeting the above criteria (e.g. dehydration not observed)

**Dysentery (B)**

Level 1 Bloody diarrhea with one of the following:

- Isolation of *Shigella* from stools
- Identification of *E.histolytica* trophozoites in stools

Level 2A Bloody diarrhea with all of the following:

- Fever
- Gripping abdominal pain
- Tenesmus and/or rectal prolapse

Level 3 Death associated with bloody diarrhea not meeting the above criteria

**Malaria (B)**

Level 1 Thick malaria smear ≥5,000 parasites/microL or ≥150 parasites/200 wbcs

PLUS one of the following:

- Axillary or oral temperature ≥38.5⁰C
- Rectal temperature ≥39⁰C

Level 2 Both of the following:

- Rapid diagnostic test positive for malaria
- Strong clinical and epidemiological support for the diagnosis of malaria

Level 3B One of the following:

- Thick malaria smear ≥5,000 parasites/microL or ≥150 parasites/200 wbcs
- Fever not meeting the above criteria
- Lack of clinical and epidemiological support for determination of malaria (i.e. the presence of parasitemia alone is not sufficient for a diagnosis of death due to malaria)

**Pelvic Inflammatory Disease (B)**

Level 1 Each of the following:

- Lower abominal tenderness
- Unilateral or bilateral adnexal tenderness
- Cervical motion tenderness

PLUS one or more of the following:

- Abnormal cervical or vaginal discharge
- Axillary or oral temperature ≥38.5^o^C
- Rectal temperature ≥39^o^C
- Elevated ESR
- Elevated C-reactive protein
- Laboratory documentation of cervical infection due to N.gonorrhea or C.trachomatis

Level 3 Clinical diagnosis of PID failing to meet the above criteria

Excludes: Death within six weeks of puerperal sepsis or septic abortion (assign to maternal deaths)

Excludes: Deaths certified as being from ectopic pregnancy or appendicitis or other gastrointestinal causes of peritonitis (assign to these causes)

**Pneumonia (A)**

Level 1 Chest x-ray consistent with pneumonia (primary end-point consolidation or pleural effusion or other consolidation/infiltration)

PLUS two or more of the following:

- Respiratory rate >20 breaths/minute
- Abnormal breath sounds/Auscultations (i.e., signs of consolidation, crepitations)
- Axillary or oral temperature ≥38.5^0^  (rectal temperature ≥39^o^C)

Level 2A Two or more of the following:

- Respiratory rate >20/minute
- Abnormal breath sounds/Auscultations (i.e., signs of consolidation, crepitations)
- Axillary or oral temperature ≥38.5^0^ (rectal temperature ≥39^o^C)

Level 3 Death associated with cough, dyspnoea, and an acute febrile illness but not meeting the above

criteria

**Pulmonary tuberculosis (A)**

Level 1 Both of the following:

- Clinical history consistent with active pulmonary tuberculosis (e.g. cough, hemoptysis, weight loss, breathlessness, fever) during terminal illness
- Negative for HIV on ELISA test or two negative HIV rapid tests

PLUS one of the following:

- Two sputum smears positive for acid fast bacilli or culture positive for M.tuberculosis
- AFB smear positive or culture positive from material from other site (e.g. lymph nodes)

Level 2A Clinical history consistent with active pulmonary tuberculosis (e.g. cough, hemoptysis, weight loss, breathlessness, fever) during terminal illness

PLUS one of the following:

- Two sputum smears positive for acid fast bacilli or culture positive for M.tuberculosis
- AFB smear positive or culture positive from material from other site (e.g. lymph nodes)

Note: Level 2 diagnosis is acceptable only in areas with low HIV prevalence where HIV testing is not consistent with standard of care.

Level 3 Clinical history consistent with active pulmonary tuberculosis in the absence of laboratory

confirmation

Excludes chronic lung disease as a result of tuberculosis

**Other Infectious Diseases (B) (Residual Category)**

None of the above diagnoses.

Level 1 Laboratory confirmation of the infecting agent

Level 2 Clinical but not laboratory evidence

**II. Cancers**

**Breast Cancer (A)**

Level 1 One of the following:

- Operative specimen with histological confirmation
- Biopsy/fine needle aspiration cytology

Level 2A Both of the following:

- Mammography diagnosis
- Imaging evidence of metastases in bone, lung, etc. based on CT scan/MRI/x-rays

Level 2B Patient under treatment from a recognised cancer hospital or cancer unit for breast cancer in cases where the basis for the initial diagnosis is no longer available

Level 3 Clinical diagnosis of breast cancer in absence of above confirmation

**Cervical Cancer (A)**

Level 1 One of the following

- Biopsy
- Pap smear and clinical diagnosis of cervical cancer

Level 2A Visualization of cervical tumor by any means

Level 2B Patient under treatment from a recognised cancer hospital or cancer unit for cervical cancer in cases where the basis for the initial diagnosis is no longer available

**Colorectal Cancer (A)**

Level 1 One of the following:

- Operative specimen with histological confirmation
- Colonoscopy report with biopsy

Level 2B Patient under treatment from a recognised cancer hospital or cancer unit for colorectal cancer in cases where the basis for the initial diagnosis is no longer available OR

One of the following in isolation:

- - - - Colonoscopy report (gross)
      - CT/MRI evidence
      - Barium contrast radiology report
      - Surgeon’s report of laparotomy

Level 3 Clinical or imaging diagnosis in the absence of any visualisation of a tumor

**Esophageal Cancer (B)**

Level 1 One of the following:

- Operative specimen with histological confirmation
- Endoscopy report with biopsy

Level 2A One of the following:

- Endoscopy report (gross)
- CT/MRI evidence
- Barium contrast radiology report

Level 2B Patient under treatment from a recognised cancer hospital or cancer unit for esophageal cancer in cases where the basis for the initial diagnosis is no longer available

Level 3 Clinical: sensation of food or liquid sticking in the gullet in absence of further confirmation

**Leukemia (B)**

Level 1 Positive test on bone marrow biopsy

Level 2B Patient under treatment from a recognised cancer hospital or cancer unit for leukemia in cases where the basis for the initial diagnosis is no longer available OR

- Blood smear diagnosis in absence of marrow biopsy

Level 3 Clinical diagnosis in absence of hematological confirmation

**Primary Liver Cancer (A)**

Level 1 One of the following:

- Operative specimen with histological confirmation
- Biopsy specimen/fine needle aspiration cytology

Level 2B Patient under treatment from a recognised cancer hospital or cancer unit for primary hepatic cancer in cases where the basis for the initial diagnosis is no longer available OR

Level 3 One or more hepatic masses (clinical or ultrasound)

**Lung Cancer (A)**

Level 1 One of the following:

- Operative specimen with histological confirmation
- Bronchoscopy report with biopsy
- Sputum cytology/fine needle aspiration cytology

Level 2A One of the following:

- Imaging report (CT scan, MRI)
- Bronchoscopy diagnosis
- Chest radiograph with a single large mass evident

Level 2B Patient under treatment from a recognised cancer hospital or cancer unit for lung cancer in cases where the basis for the initial diagnosis is no longer available

Level 3 Chest radiograph with more than one mass OR Clinical diagnosis

**Lymphomas (B)**

Level 1 Positive test on lymph node biopsy

Level 2B Patient under treatment from a recognised cancer hospital or cancer unit for lymphoma in cases where the basis for the initial diagnosis is no longer available

Level 3 Clinical diagnosis in absence of biopsy

**Mouth/Oropharynx Cancer (B)**

Level 1 Operative/biopsy specimen with histological confirmation

Level 2B Patient under treatment from a recognised cancer hospital or cancer unit for oropharyngeal cancer in cases where the basis for the initial diagnosis is no longer available OR visualisation of the cancer by a cancer specialist in the absence of a biopsy

Level 3 Clinical report not meeting the above criteria

**Ovarian Cancer (B)**

Level 1 Operative specimen with histological confirmation

Level 2B Patient under treatment from a recognised cancer hospital or cancer unit for ovarian cancer in cases where the basis for the initial diagnosis is no longer available

Level 3 Clinical or imaging diagnosis in absence of histology

**Prostate Cancer (B)**

Level 1 Operative/biopsy specimen with histological confirmation

Level 2A Elevated PSA

PLUS one of the following:

- - - - Transrectal ultrasound morphology
      - Evidence of metastases

Level 2B Patient under treatment from a recognised cancer hospital or cancer unit for prostate cancer in cases where the basis for the initial diagnosis is no longer available OR Clinical diagnosis (nodular prostate) with metastases but no PSA test result

Level 3 Clinical diagnosis (nodular prostate) without metastases without PSA test result

**Stomach Cancer (A)**

Level 1 One of the following:

- Operative specimen with histological confirmation
- Endoscopy report with biopsy

Level 2A One of the following:

- Endoscopy report (gross)
- CT/MRI evidence
- Barium contrast radiology report

Level 2B Patient under treatment from a recognised cancer hospital or cancer unit for stomach cancer in cases where the basis for the initial diagnosis is no longer available

Level 3 Clinical or imaging diagnosis in absence of histology

**Uterine Cancer (B)**

Level 1 One of the following:

- Operative specimen with histological confirmation
- Biopsy
- Pap smear and clinical diagnosis of uterine cancer

Level 2A One of the following:

- Colposcopy/hysteroscopy report (gross)
- CT/MRI evidence of primary mass or lesion in the uterus

Level 2B Patient under treatment from a recognised cancer hospital or cancer unit for uterine cancer in cases where the basis for the initial diagnosis is no longer available

Level 3 Clinical diagnosis

**Other Defined Cancers (B) (Residual Category)**

Cancer from sites other than the above. Exclude any cancer deaths with clinical diagnosis only.

Level 1 Operative specimen with histological confirmation

Level 2B Patient under treatment from a recognised cancer hospital or cancer unit for cancer from a specific site other than the above in cases where the basis for the initial diagnosis is no longer available

**III. Adult Non-communicable**

**Asthma (B)**

Level 1 Both of the following:

- Evidence from spirometry or serial peak flow measurement of reversible airway obstruction > 20% AND
- Status asthmaticus: severe case of prolonged wheezing leading to death as assessed by a physician

Level 2A Both of the following:

- Status asthmaticus: recent onset of an episode of severe dyspnoea associated with wheeze leading to death
- Past history of episodic breathlessness and wheeze diagnosed as asthma

Level 3 Severe dyspnoea and wheeze leading to death in absence of clinical or laboratory evidence of

Asthma

**Cardiomyopathy (B) (as categorized under “Inflammatory Heart Disease”)**

Level 1 Echocardiograph showing a globally hypokinetic, dilated heart in the presence of CCF and in the absence of ischemia, valvular defects, or pericardial disease

Level 3 Radiographic and clinical evidence of a large, dilated heart with CCF in the absence of an

echocardiograph

**Cirrhosis (A)**

Level 1 Liver biopsy

Level 2A One of the following:

- Chronic liver failure supported by evidence of abnormal liver function tests and characteristic abnormalities on imaging
- Bleeding from confirmed oesophageal varices

Level 3 Clinical evidence of liver failure or upper GI bleeding without supporting laboratory confirmation or

imaging

**COPD (A)**

Level 1 Diagnosis established by one of:

- Spirometry diagnosis FEVI<70% with no response to bronchodilators
- Chest x-ray features hyperinflation, flat hemi-diaphragms, reduced peripheral vascular markings, presence of bullae in conjunction with clinical features of COPD

AND Terminal illness due to one of:

- Pneumonia
- Cor pulmonale
- Respiratory failure

Level 2B Diagnosis established by the following criteria:

- Productive cough and breathlessness for 3 or more months of the year for a minimum of 2 successive years

Level 3 Clinical diagnosis of COPD not meeting the above criteria

**Dementia (B)**

Level 1 One of the following:

- Clinical diagnosis of dementia by a neurologist, psychiatrist, or psychologist
- CT/MRI based evidence of multiple cerebral infarcts and clear history of progressive impairment of cognition in clear consciousness, manifested by memory loss, etc.

Level 2B Clinical diagnosis of dementia by a general practicioner

Level 3 Symptom history only; non-medical diagnosis

**Diabetes with Coma (B)**

Level 1 Both of the following:

- - - - Gold standard diagnosis of diabetes (see Appendix A)
      - Terminal features of diabetic ketoacidosis/hyperosmolar non ketotic coma

Level 3 Both of the following:

- Clinical history of diabetes not meeting the above laboratory criteria
- Terminal features of diabetic ketoacidosis/hyperosmolar non ketotic coma

**Diabetes with Renal Failure (B)**

Level 1 Gold standard diagnosis of diabetes (see Appendix A) plus gold standard diagnosis of renal failure

Level 3 Both of the following:

- Clinical history of diabetes not meeting the above laboratory criteria
- Biochemical evidence of renal failure

**Diabetes with Skin Infection/Sepsis (B)**

Level 1 Both of the following:

- Gold standard diagnosis of diabetes (see Appendix A)
- Diabetic foot ulcers, sacral ulcers, or other skin lesions characteristic of diabetes with septic shock (see Appendix B)

Level 3 Both of the following:

- Clinical history of diabetes not meeting the above laboratory criteria
- Presence of foot ulcers, sacral ulcers, or other skin lesions suggestive of diabetes

**Bacterial Endocarditis (as categorized under “Inflammatory Heart Disease”)**

Level 1 Both of the following:

- Echocardiagraph showing vegetations on heart valves
- Evidence of bacteremia from blood cultures

Level 2A At least three of the four following:

- Embolic phenomena (petechiae, retinal haemorrhages etc.)
- Evidence of bacteremia from blood cultures
- Cardiac murmur OR recent invasive procedures OR history of drug abuse parenterally
- Axillary or oral temperature ≥38.5⁰C (rectal temperature ≥39⁰C)

Level 3 Cardiac murmur with fever

Epilepsy (B)

Level 1 Both of the following

- Status epilepticus: repeated seizures leading to death either from airway obstruction or brain damage observed and documented by a clinician, in the absence of evidence of an underlying cause such as: intracranial space occupying lesion, cerebrovascular disease, connective tissue disorders, metabolic derangements, or CNS infections
- Past history of seizures

Level 3 Seizures before death not meeting the above criteria

IHD – Acute Myocardial Infarction (A)

Level 1 Evidence of acute MI within 3 months preceding death based upon one or more of the following:

- Cardiac perfusion scan
- ECG changes
- Documented history of CABG or PTCA or stenting
- Coronary angiography
- Enzyme changes (any troponin elevation or CK-MB isoenzyme elevation>2 times the upper limit of normal) in the context of myocardial ischemia

Level 2A Clinical evidence of the following:

- Sudden death within six hours of the onset of characteristic shock (see Appendix B) and chest pain when the case has been witnessed by a physician

Level 3 Sudden death associated with chest pain not meeting Level 2 criteria

IHD – Congestive Heart Failure (A)

Level 1 Both of the following:

- Documented history of ischaemic/hypertensive heart disease including ECG changes
- Radiological evidence of pulmonary congestion

Level 3 Clinical diagnosis of congestive heart failure

**Pericarditis (B) (as categorized under “Inflammatory Heart Disease”)**

Level 1 One of the following:

- ECG changes and evidence of pericardial effusion on echocardiography
- Aspiration of fluid from the pericardium

Level 3 Clinical diagnosis of pericarditis

**Renal Failure (A)**

Level 1 No diabetes or other primary clinically identified cause of death

PLUS one of the following:

- - - - Renal biopsy showing evidence of glomerular/interstitial/tubular disease
      - Elevated blood urea nitrogen and/or creatinine

Level 3 Meets the above criteria but diabetes not excluded

**Stroke (A)**

Level 1 Both of the following:

- CT scan/MRI
  - - - Sudden onset of paralysis, coma

Level 2A Within the 28 days prior to death, rapidly developing signs of a focal or global loss of cerebral function lasting more than 24 hours (or leading to death) with no apparent cause other than that of vascular origin

**Other Specified Cardiovascular Diseases (B) (Residual Category)**

None of the above diagnoses. Exclude poorly defined conditions, e.g. “cardiac failure.”

**Other Specified Digestive Diseases (B) (Residual Category)**

None of the above diagnoses. Excludes poorly defined conditions, e.g. melena.

**Other Non-communicable diseases (B) (Residual Category)**

None of the above diagnoses.

**IV. Maternal**

A maternal death is the death of a woman during pregnancy or within 6 weeks of either abortion or birth. It is a death from any cause related to or aggravated by the pregnancy or its management, but not from accidental or incidental causes.

For the causes listed below, the specific cause must be confirmed by a physician or registered midwife or by laboratory tests.

**Anemia (B)**

Level 1 Both of the following:

- - - - Hemoglobin <3 AND
      - Clinical diagnosis of congestive heart failure

**Hemorrhage (B)**

Level 1 Shock (see Appendix B) following excessive blood loss from one of the following:

- Antepartum hemorrhage due either to placenta praevia or placental separation (abruption)
- Primary postpartum haemorrhage (within 24 hours of vaginal delivery) associated with the measured loss of 500mls of blood or more
- Secondary postpartum haemorrhage (more than 24 hours after delivery)
- Spontaneous or medically induced abortion

Level 2A Level 2A Shock (see Appendix B) following excessive blood loss from one of the following:

- Antepartum hemorrhage due either to placenta previa or placental separation (abruption)
- Primary PPH (within 24 hours of vaginal delivery) associated with significant blood loss clinically assessed as requiring transfusion or having received transfusion
- Secondary PPH (more than 24 hours after delivery)
- Spontaneous or medically induced abortion

**Sepsis (B)**

Level 1 All of the following:

- Pyrexia ≥38^o^C
- Lower abdominal tenderness and pain
- Offensive discharge
- Clinical evidence of Level 1 shock (see Appendix B)
- Positive blood culture

Level 2A All of the following:

- Pyrexia ≥38^o^C
- Lower abdominal tenderness and pain
- Offensive discharge
- Clinical evidence of Level 2 shock (see Appendix B)

**Eclampsia (B)**

Level 1 All of the following:

- BP ≥140/90 mm Hg at 20 weeks gestation or later
- Albuminuria ≥ 3+
- Seizures leading to death
- No history of epilepsy or other reason for seizures (e.g., malaria or other acute encephalopathy)

Level 2A All of the following:

- BP >=140/90 at 20 weeks gestation or later
- Seizures leading to death
- No history of epilepsy or other reason for seizures (e.g., malaria or other acute encephalopathy)

Note: Excludes epilepsy and hypertension from other specific causes

**Obstructed Labor (B)**

Level 1 Diagnosis established by all of the following:

- Prolonged labor (> 12 hours)
- Failure of the presenting part to descend
- Failure of the cervix to dilate fully
- Excessive moulding if the head is the presenting part

PLUS terminal illness due to one of:

- - - - Surgical diagnosis of uterine rupture
      - Level 1 Sepsis

Level 2A All of the following:

- Prolonged labor (>12 hours)
- Failure of presenting part to descend
- Failure of the cervix to fully dilate
- Excessive moulding if the head is the presenting part

PLUS terminal illness due to one of:

- - - - Uterine rupture established by clinical examination
      - Level 2A Sepsis (see Appendix B)

**Other Defined Causes of Death as a Consequence of Pregnancy (B) (Residual Category)**

None of the above diagnoses. Excludes death as a consequence of pregnancy where the immediate cause is undefined.

**V. Injuries**

The following causes are ALWAYS considered accidental: Bite of Venomous Animal, Drowning, Falls, Fires, Poisonings, Road Traffic. They are NEVER overlapping with Homicide or Suicide. If Homicide is indicated, it is the ONLY cause. If Suicide is indicated, it is the ONLY cause.

**Bite of Venomous Animal (B)**

Level 1 Third party written accounts: police report, coroner’s/autopsy report, hospital record, newspaper

account

**Drowning (B)**

Level 1 Third party written accounts: police reports, coroner’s/autopsy report, hospital record, newspaper

account

**Falls (B)**

Level 1 Third party written accounts: police reports, coroner’s/autopsy, hospital record, newspaper account

**Fires (B)**

Level 1 Third party written accounts: police reports, coroner’s/autopsy, hospital record, newspaper account

**Poisonings (B)**

Level 1 Third party written accounts: police reports, coroner’s/autopsy, hospital record, newspaper account

**Road Traffic (B)**

Level 1 Third party written accounts: police reports, coroner’s/autopsy, hospital record, newspaper account

**Homicide (B)**

Level 1 Third party written accounts: police reports, coroner’s/autopsy, hospital record, newspaper account

**Suicide (B)**

Level 1 Third party written accounts: police reports, coroner’s/autopsy, hospital record, newspaper account

**Other Injuries (B) (Residual category)**

None of the above diagnoses.

Level 1 Third party written accounts: police reports, coroner’s/autopsy, hospital record, newspaper account

**Appendix A. Co-morbid Conditions**

If one of these conditions is present in addition to the primary gold standard cause of death, it should be noted in the data.

Gold standard conditions associated with the underlying cause of death and the following two conditions should be included as co-morbid conditions.

**Diabetes**

Screening for Diabetes in a healthy person:

Level 1 Screening one of:

- Fasting glucose ≥7.0 mmol/L (≥126 mg/dl)
- Oral glucose tolerance test, 2 hour glucose ≥11.1 mmol/L (≥200 mg/dl)
- HbA_1c_>6.5 mg/dl

Screening for diabetes in a sick person admitted to the hospital:

Level 1 Hospitalization for a complication of diabetes and no documented history of diabetes:

- Random glucose ≥11.1 mmol/L (≥200 mg/dl) on at least two occasions not influenced by recent meal or intravenous glucose

Hospitalization for a complication of diabetes a documented history of diabetes:

- Random glucose ≥11.1 mmol/L (≥200 mg/dl) on at least one occasion not influenced by recent meal or intravenous glucose

**Hypertension**

Level 1 One of the following:

- Systolic BP ≥ 140 mm Hg
- Diastolic BP ≥ 90 mm Hg

**Appendix B. Common Terminal Condition(s)**

This appendix is meant to detail conditions that are common across multiple priority gold standard causes of death, to help clarify diagnosis of such conditions. Shock is the only common terminal condition listed at this time.

**Shock**

Level 1 Three of the following:

- Obtunded
- Heart rate > 100
- Respiratory rate > 22
- Hypotension (systolic BP <100 mm Hg or a 30 mm fall in baseline BP
- Urine output <0.5 mL/Kg/hour

Level 2A Clinical diagnosis of shock
