# Supplementary material for: Population Health Metrics Research Consortium gold standard verbal autopsy validation study: design, implementation, and development of analysis datasets
Source: Popul Health Metr. 2011 Aug 4;9:27. doi: 10.1186/1478-7954-9-27 (PMC3160920; doi:10.1186/1478-7954-9-27)
Supplement: Additional file 7 — Gold standard (GS) definitions in the PHMRC study for children/neonates. [file 1478-7954-9-27-S7.DOC]

**Neonatal and Child Gold Standard Diagnoses**

Level 1 = Diagnosis of a particular condition with the highest level of certainty possible for that condition, consisting of either an appropriate laboratory test or x-ray with positive findings and/or medically observed and documented appropriate illness sign(s)

Level 2A = Diagnosis of a particular condition with a high level of certainty, consisting of medically observed and documented appropriate illness sign(s).

Level 2B= Diagnosis of a particular condition with reasonable certainty but not meeting Level 1 or Level 2A criteria: this category was developed especially for cancer and HIV diagnoses where records are not available

Level 3 = Cases which would be considered for a gold standard diagnosis but do not meet gold standard criteria: “possible gold standard cases.” This level is designed to exclude possible gold standard cases from the residual categories; no VAs should be collected for any level 3 causes of death.

**Notes:**

1. To be acceptable, illness signs must be observed and documented in a medical record by a physician, clinical officer or, in the case of stillbirths, preterm delivery or birth asphyxia, by a midwife.

2. Level 1 diagnosis should be the standard used for all gold standard cases. Only if it proves impossible to gather enough cases of a particular condition is it allowable to use the Level 2 diagnosis. For all causes, an autopsy report would be acceptable as a gold standard confirmation. (Details are provided below.)

3. Residual Categories (Appendix A): In addition to the priority gold standard causes listed below, the data analysis method requires us to collect a sample of deaths from non-priority causes. These deaths will be grouped into residual categories. Thus, the residual categories will include deaths that occur from non-priority causes, clustered according to Global Burden of Disease cause spans to allow for a balanced distribution of residual causes in the data. The purpose of the Level 3 diagnosis is to prevent possible gold standard deaths from inclusion in the residual categories. If there is a suspicion that the death may be due to one of the priority gold standards, the death does not qualify for the residual category and should be excluded.

4. Several pediatric conditions commonly occur together and may have additive or synergistic effects in causing death. These “co-morbid” conditions are listed at the end of the neonatal and childhood sections.

NEONATAL (Age < 28 days)

1. Neonatal Conditions

Birth Asphyxia (A)

Level 1 Each of the following:

- Failure both to breathe spontaneously and to cry at birth
- No major congenital abnormality
- Not a stillbirth (one or more signs of life at birth like pulse or movement)

PLUS one of the following in the 24 hours after birth:

- - - - Not feeding
      - Hypotonia
      - Seizures
      - Needed and failed resuscitation at birth

Congenital Malformation (A)

Level 1 Both of the following:

- Congenital malformation that is externally visible OR established by an imaging study
- Congenital malformation that is assessed by the study physician to have been the cause of death

*Examples:* Esophageal atresia; Gastroschisis; Hydrocephalus; Hypertrophic pyloric stenosis; Imperforate anus; Intestinal obstruction; Omphalocele

Level 3 Death assessed to have been caused by an internal congenital malformation that was not established by an imaging study

Meningitis (A) (as categorized under “Serious Infection”)

Level 1 Positive lumbar puncture, defined as one of the following:

- Any bacteria seen on gram stain of CSF
- >20 leukocytes/mm^3^ CSF with >80% PMNs
- Positive latex agglutination test of CSF
- Positive CSF culture

Level 3 Clinical diagnosis of meningitis with no lumbar puncture performed

Neonatal Tetanus (A)

Level 1 All of the following:

- Age at illness onset >2 days
- Not able to open mouth
- Rigidity

PLUS one of the following:

- Opisthotonus
- Spasms
- Convulsions

Pneumonia (A) (as categorized under “Serious Infection”)

Level 1 Chest x-ray showing primary end-point consolidation, pleural effusion or other consolidation/infiltration

PLUS two or more of the following:

- Respiratory rate >70/minute
- Severe lower chest indrawing
- Abnormal breath sounds (i.e., grunting, decreased breath sounds, crepitations)
- Rectal temperature >38^0^C or <36^0^C
- Oral or Axillary temperature >37.5^0^C or <35.5^0^C

Level 3 An acute febrile illness or hypothermia with cough and dyspnea, but not meeting the above criteria

Preterm Delivery (without Respiratory Distress Syndrome) (A)

Level 1 Not a stillbirth

PLUS one of the following:

- Birth at <33 weeks gestation (based on birth date minus the mother’s reported date of her last menstrual period)
- A physician or clinical officer’s Ballard gestational age assessment of <33 weeks (for hospital births)

PLUS one of the following:

- Chest x-ray negative for the characteristic “ground glass” appearance of RDS
- Death from another medically documented neonatal condition

Level 3 Meet all the above criteria, except gestational age = 33-36 weeks

**Preterm Delivery with Respiratory distress syndrome (RDS) (B)**

Level 1 Chest x-ray positive for characteristic “ground glass” appearance

PLUS one of the following:

- - - - - Preterm delivery: Birth at <33 weeks gestation, based on:

Birth date minus the mother’s reported date of her last menstrual period

A physician or clinical officer’s Ballard gestational age assessment <33 weeks

- - - - - Not preterm delivery: Birth at 33-36 weeks gestation, based on:

Birth date minus the mother’s reported date of her last menstrual period

A physician or clinical officer’s Ballard gestational age assessment 33-36 weeks

PLUS two or more of the following:

- - - - - Respiratory rate >70/minute
        - Central cyanosis (dusky, bluish lips or mucus membranes)
        - Severe lower chest wall indrawing
        - Grunting
        - Nasal flaring

Level 3 Death of a newborn <37 weeks gestation that breathed spontaneously at birth, from a non-hypothermic illness with tachypnea but not meeting the above criteria

Sepsis (A) (as categorized under “Serious Infection”)

Level 1 Positive laboratory criteria, including one of the following:

- Positive blood culture
- I:T (immature:total) neutrophil ratio >0.2

PLUS one of the following:

- Rectal temperature >38^o^C or <36^0^C
  - - - Oral or Axillary temperature >37.5^0^C or <35.5^0^C

PLUS one of the following:

- Irritability/agitated
- Abnormally sleepy, difficult to wake, lethargic or reduced to no spontaneous movement
- Unconscious
- Absent or weak cry
- Absent or weak suck
- Respiratory distress: respiratory rate >70/minute, severe lower chest indrawing, or grunting
- Mottled/cyanotic and cool extremities
- Reduced blood pressure

Level 3: Children who meet the Level 1 criteria but for whom either (1) both a blood culture and an I:T neutrophil ratio were obtained and both were negative, or (2) only an I:T neutrophil ratio was obtained and it was negative.

**Sepsis with local bacterial infection (B)**

Level 1 Level 1 Sepsis (A)

PLUS one of the following:

- Red umbilicus extending to the skin
- Purulent umbilicus
- Skin pustules
- Bullae
- Ulcer
- Furuncle (boil)
- Cellulitis

Level 3 Level 3 Sepsis (A) PLUS one of the above listed local bacterial infections

Stillbirth (A)

Level 1 Birth of a fetus without any sign of life, including movement of voluntary muscles, spontaneous breathing, cry or pulsation of the umbilical cord

PLUS one of the following:

- Birth at >28 weeks gestation (based on birth date minus the mother’s reported date of her last menstrual period)
- Birth weight >1,000 grams

Level 3 Birth of a fetus without any sign of life (as above)

PLUS one of the following:

- Birth at 22-27 weeks gestation (based on birth date minus the mother’s reported date of her last menstrual period)
- Birth weight 500-999 grams

II. Neonatal Co-morbid Conditions

Preterm delivery (without RDS) AND Birth asphyxia (B)

Level 1 Level 1 Preterm delivery (without RDS) PLUS Level 1 Birth asphyxia

Level 3 Level 3 Preterm delivery (without RDS) PLUS Level 3 Birth asphyxia

Preterm delivery (with or without RDS) AND Sepsis (B)

Level 1 Level 1 Preterm delivery (with or without RDS) PLUS Level 1 Sepsis

Level 3 Level 3 Preterm delivery (with or without RDS) PLUS Level 3 Sepsis

Preterm delivery (without RDS) AND Sepsis AND Birth asphyxia (B)

Level 1 Level 1 Preterm delivery (without RDS) PLUS Level 1 Sepsis AND Level 1 Birth asphyxia

Level 3 Level 3 Preterm delivery (without RDS) PLUS Level 3 Sepsis AND Level 3 Birth asphyxia

CHILDHOOD (Age ≥ 28 days and < 12 years)

I. Childhood Infectious Diseases

AIDS (A)

Level 1 No Level 1 TB

PLUS one of the following:

- Age at death <18 months and positive child HIV DNA PCR
- Age at death >18 months and positive child ELISA

PLUS one of the following:

- Age at death <12 months and CD4 count <20%
- Age at death 12-59 months and CD4 count <15%
- Age at death <18 months and total lymphocyte count <2500/mm^3^
- Age at death >18 months and total lymphocyte count <1500/mm^3^

       PLUS one of the following:

- Unexplained severe wasting or severe malnutrition not adequately responding to standard therapy
- Pneumocystis pneumonia
- Recurrent severe presumed bacterial infections (e.g. empyema, pyomyositis, bone or joint infection, meningitis, but excluding pneumonia)
- Chronic herpes simplex infection (orolabial or cutaneous of more than one month’s duration)
- Kaposi’s sarcorma
- Oesophageal candidiasis
- CNS toxoplasmosis (outside the neonatal period)
- HIV encephalopathy

Level 2B Patient receiving treatment with ARV where the basis for the initial diagnosis is no longer available

Level 3 Both of the following:

- No Level 1, 2 or 3 TB
- Clinical evidence of AIDS

PLUS one or both of the following:

- No HIV testing
- No CD4 testing AND no total lymphocyte testing

AIDS with Tuberculosis (A)

Level 1 Level 1 TB

PLUS one of the following:

- Age at death <18 months and positive child HIV DNA PCR
- Age at death >18 months and positive child ELISA

PLUS one of the following:

- Age at death <12 months and CD4 count <20%
- Age at death 12-59 months and CD4 count <15%
- Age at death <18 months and total lymphocyte count <2500/mm^3^
- Age at death >18 months and total lymphocyte count <1500/mm^3^

Level 3 Both of the following:

- Level 3 TB: Clinical history consistent with active pulmonary TB without laboratory or radiographic evidence
- HIV positive OR CD4 or total lymphocyte count as above

Diarrhea (A)

Level 1 All of the following:

- Reported liquid or watery or loose stools 3+ times a day for at least 1 day
- Dehydration
  - - - Observed liquid or semi liquid or watery stools

Level 3 Reported liquid, watery or loose stools

PLUS one or both of the following:

- No dehydration
- No observed liquid, semi liquid or watery stools

Dysentery (A)

Level 1 All of the following:

- Reported liquid or watery or loose stools 3+ times a day
- Observed gross blood in the stools
- Fever

PLUS one of the following:

- Documented pediatric septic shock
- Documented hemolytic uremic syndrome
- Documented renal failure

Level 3 A febrile illness with observed gross blood in the stools, but without documented septic shock, hemolytic uremic syndrome or renal failure

Encephalitis (B)

Level 1 Positive lumbar puncture, defined as all of the following:

- >10 leukocytes/ mm^3^ and >50% lymphocytes
- No bacteria seen on gram stain of CSF
- No bacteria seen on CSF culture (if performed)
- Negative latex agglutination test of CSF (if performed)

Level 3 Clinical diagnosis of encephalitis with no lumber puncture performed

Hemorrhagic Fever (B)

Level 1 Evidence of internal bleeding

PLUS one of the following:

- Rectal temperature >38^o^C or <36^0^C
- Oral or Axillary temperature >37.5^0^C or <35.5^0^C

Level 3 Both of the following:

- Evidence of internal bleeding
- Fever or hypothermia not meeting the above criteria

Malaria (A)

Level 1 Thick malaria smear >10,000 parasites/microL

PLUS one of the following:

- Rectal temperature >38^o^C
- Oral or Axillary temperature >37.5^o^C

Level 2A Rapid diagnostic test positive

PLUS one of the following:

- Rectal temperature >38^o^C
- Oral or Axillary temperature >37.5^o^C

Level 3 A febrile illness with a positive thick malaria smear

PLUS one of the following:

- Thick malaria smear <10,000 parasites/microl
- Fever not meeting the above criteria

Measles (B)

Level 1 Positive measles-specific IgM antibodies

Level 2 A One of the following:

- Koplik spots
- Blotchy or confluent maculopapular rash on the face alone or most prominent on the face

PLUS one of the following

- Rectal temperature >38^0^C
- Oral or Axillary temperature >37.5^0^C

PLUS at least one of the following:

- Cough
- Coryza
- Conjunctivitis

PLUS death from diarrhea or pneumonia complications within 30 days of the acute rash illness

Level 3 An acute illness with rash and fever, but not meeting the above criteria

Meningitis (B)

Level 1 Positive lumbar puncture, defined as one of the following:

- Bacteria on gram stain of CSF
- >100 leukocytes/ mm^3^ CSF with >80% PMNs
- Positive latex agglutination test of CSF
- Positive CSF culture

Level 3 Clinical diagnosis of meningitis with no lumbar puncture performed

**Pertussis (B)**

Level 1 Both of the following:

- Culture positive for *B. Pertussis*
- An acute cough illness of any duration

OR both of the following:

- PCR positive for pertussis
- Meets the CDC clinical case definition (see below)

OR both of the following:

- Epidemiological link to a case confirmed by culture or PCR
- Meets the CDC clinical case definition (see below)

Level 2A Case meets the CDC clinical case definition: Cough >2 weeks

PLUS one of the following:

- Paroxysms of coughing
- Inspiratory “whoop”
- Posttusive vomiting

Level 3 An illness with prolonged cough not meeting the above criteria

Pneumonia (A)

Level 1 Chest x-ray consistent with pneumonia (primary end-point consolidation or pleural effusion or other consolidation/infiltration*)

PLUS any 2 or more of the following:

- Respiratory rate >50/minute
- Lower chest indrawing
- Abnormal breath sounds/auscultations (i.e., signs of consolidation, crepitations)
- Rectal temperature >38^0^ or oral/axillary temperature >37.5^0^

Level 2A Any 2 or more of the following:

- Respiratory rate >50/minute
- Lower chest indrawing
- Abnormal breath sounds/auscultations (i.e., signs of consolidation, crepitations)
- Rectal temperature >38^0^ or oral/axillary temperature >37.5^0^

Note: A negative chest x-ray does not disqualify a level 2A pneumonia diagnosis.

These symptoms capture acute lower respiratory infections but most will be pneumonia

Level 3 An acute febrile illness with cough and dyspnoea, but not meeting the above criteria

*As indicated in the WHO Standardization of interpretation of chest radiographs for the diagnosis of pneumonia in children. WHO, Pneumonia Vaccine Trial Investigators’ Group, 2001, Annex 4.

Sepsis (B)

Level 1 Positive laboratory criteria, defined as one of the following:

- Positive blood culture
- I:T (immature:total) neutrophil ratio >0.2

PLUS one of the following:

- Rectal temperature >38^o^C or <36^o^C
- Oral or Axillary temperature >37.5^o^C or <35.5^o^C

PLUS one of the following:

- Irritability/agitated
- Abnormally sleepy, difficult to wake, lethargic or reduced to no spontaneous movement
- Unconscious
- Respiratory distress: respiratory rate >50/minute or lower chest indrawing or grunting
- Mottled/cyanotic and cool extremities
- Reduced blood pressure

Level 2A Meets clinical criteria, including one of the following:

- Rectal temperature >38^o^C or <36^o^C
- Oral or Axillary temperature >37.5^o^C or <35.5^o^C

PLUS one of the following:

- Irritability/agitated
- Abnormally sleepy, difficult to wake, lethargic or reduced to no spontaneous movement
- Unconscious
- Respiratory distress: respiratory rate >50/minute or lower chest indrawing or grunting
- Mottled/cyanotic and cool extremities
- Reduced blood pressure

Note: A negative blood culture alone (when an I:T neutrophil ratio was not obtained) does not disqualify a level 2A sepsis diagnosis.

Level 3 Children who meet the Level 1 or Level 2 criteria but for whom either (1) both a blood culture and an I:T neutrophil ratio were obtained and both were negative, or (2) only an I:T neutrophil ratio was obtained and was negative.

**Sepsis with local bacterial infection (B)**

Level 1 Level 1 Sepsis

PLUS one of the following:

- Skin pustules
- Bullae
- Ulcer
- Furuncle (boil)
- Cellulitis

Level 2A Level 2A Sepsis

PLUS one of the following:

- Skin pustules
- Bullae
- Ulcer
- Furuncle (boil)
- Cellulitis

Level 3 Level 3 Sepsis PLUS one of the above listed local bacterial infections

Tuberculosis (B) (only Pulmonary TB)

Level 1 One of the following:

- Culture of (sputum or gastric aspirate) specimen positive for MTB
- Two (sputum or gastric aspirate) smears positive for AFB

OR one (sputum or gastric aspirate) smear positive for AFB and chest x-ray suggestive of active TB

Level 2A    Chest x-ray suggestive of active TB

PLUS one of the following:

- Cough >2 weeks
- Known family member with TB

Level 3 Prolonged cough and a known family member with TB, but without the above laboratory or radiographic evidence

1. **Childhood Co-morbid Conditions**

**Pneumonia AND Diarrhea (B)**

Level 1 Level 1 Pneumonia PLUS Level 1 Diarrhea

Level 3 Level 3 Pneumonia PLUS Level 3 Diarrhea

**III. Childhood Injuries**

**Bite of Venomous Animal (B)**

Level 1 Third party written accounts: police report, coroner’s/autopsy, hospital record, newspaper account

**Drowning (B)**

Level 1 Third party written accounts: police reports, coroner’s/autopsy, hospital record, newspaper account

**Falls (B)**

Level 1 Third party written accounts: police reports, coroner’s/autopsy, hospital record, newspaper account

**Fires (B)**

Level 1 Third party written accounts: police reports, coroner’s/autopsy, hospital record, newspaper account

**Poisonings (B)**

Level 1 Third party written accounts: police reports, coroner’s/autopsy, hospital record, newspaper account

**Road Traffic (B)**

Level 1 Third party written accounts: police reports, coroner’s/autopsy, hospital record, newspaper account

**Violent Death (B)**

Level 1 Third party written accounts: police reports, coroner’s/autopsy, hospital record, newspaper account

**Appendix A. Childhood Residual Diseases**

**Other Childhood Infectious Diseases (B)**

None of the above diagnoses.

Level 1 Laboratory confirmation of the infecting agent

Level 2A Clinical but not laboratory evidence

**Malignant neoplasms (B)**

None of the above diagnoses. Exclude any malignant neoplasms with clinical diagnosis only.

Level 1 Histological or haematological confirmation

**Cardiovascular diseases (B)**

None of the above diagnoses. Exclude any cardiovascular diseases with clinical diagnosis only.

Level 1 Diagnosis based on appropriate cardiac function and/or imaging (e.g., echocardiograph) study

PLUS clinical findings

**Respiratory diseases (B)**

None of the above diagnoses. Exclude any respiratory diseases with clinical diagnosis only.

Level 1 Diagnosis based on appropriate lung function and/or imaging study PLUS clinical findings

**Digestive diseases (B)**

None of the above diagnoses. Exclude any digestive diseases with clinical diagnosis only.

Level 1 Diagnosis based on surgical, specimens, or biopsy findings

Level 2A Diagnosis based on visualization (endoscopic or operative findings)

**Other defined causes of child deaths (B)**

Excludes all gold standard conditions and conditions defined by other residual categories. Includes other non-communicable diseases and other injuries.
