# Supplementary material for: Population Health Metrics Research Consortium gold standard verbal autopsy validation study: design, implementation, and development of analysis datasets
Source: Popul Health Metr. 2011 Aug 4;9:27. doi: 10.1186/1478-7954-9-27 (PMC3160920; doi:10.1186/1478-7954-9-27)
Supplement: Additional file 8 — Medical data extraction form (MDEF) used to extract gold standard data for the PHMRC study. [file 1478-7954-9-27-S8.DOC]

**POPULATION HEALTH METRICS RESEARCH CONSORTIUM**

**MEDICAL RECORD DATA EXTRACTION FORM**

**SECTION 1: BASIC INFORMATION**

Name of deceased ______________________

| 1.1 Date of Admission: _ _ / _ _ / _ _ _ _  dd / mm / yyyy  1.2 Date of Birth: _ _ / _ _ / _ _ _ _  dd / mm / yyyy  1.3 Date of Death: _ _ / _ _ / _ _ _ _  dd / mm / yyyy  1.4 Age at Death: ___ ___years (if ≥ 1 yr) ___ ___ months (if < 1 yr) ___ ___ days (if < 1 month)  1.5 Date form filled: _ _ / _ _ / _ _ _ _  dd / mm / yyyy  1.6 Name of Clinician completing the form: __________________________________________   - 1. Record clinician identification number   *(if applicable)* | | | |
| --- | --- | --- | --- |
| 1.8 Location where form was filled:  *(each site can modify)* | 1 ______________  2 ______________  3 ______________ | 5 ______________  6 ______________  7 ______________ | 8 ______________  9 ­______________ |
| - 1. Residence of deceased   (*as detailed as possible*) | Address:______________________________________________________  _____________________________________________________________  _____________________________________________________________  _____________________________________________________________  *Sketch Map if necessary* | | |

SECTION 2: DETAILS ABOUT DEATH

| 2.1 Check the cause of death category:  *(select one)* | 1 Neonatal  2 Child Injuries  3 Childhood Infectious Diseases  4 Maternal  5 Adult Infectious Disease | | 6 Cancers  7 Adult Non-Communicable  8 Adult Injuries  9 Childhood residual  10 Adult residual | | 11 Neonatal  Co-morbid Conditions  12 Childhood  Co-morbid Conditions  13 Adult  Co-morbid Conditions |
| --- | --- | --- | --- | --- | --- |
| 2.2 Level of Diagnosis: | 1 Level 1 | 2 Level 2 | | 3 Level 3 (Do NOT perform VA Interview) | 9 Unable to determine from medical records |
| 2.3 Diagnosis made from: | 1 Medical Record | 2 Autopsy Report | | 3 Other  (specify): ________________________ | |

**SECTION 3: GOLD STANDARD CRITERIA FOR DIAGNOSIS**

*Directions: Using the criteria specified in the PHMRC clinical booklets for Adult and Neonatal/Child Gold Standard Diagnoses, write which of the diagnostic criteria were met by the deceased and then check the box of the final diagnosis in Section 4 below. Details of the diagnostic criteria should be recorded as much in detail as possible.*

| **Criteria** |
| --- |
| 1. |
| 2. |
| 3. |
| 4. |
| 5. |
| 6. |
| 7. |
| 8. |
| 9. |
| 10. |

**SECTION 4: NEONATAL/CHILD DEATHS ONLY**

**FINAL GOLD STANDARD DIAGNOSIS FOR NEONATAL/CHILD CAUSES**

***Directions:***  *Using information collected above, mark the appropriate diagnosis for the deceased. This page will be submitted to the data entry personnel.*

- 1. Study status

| **Neonatal**   1. Birth asphyxia (P21) …………….. 2. Congenital malformation (Q00)………………………….. 3. Sepsis with Local Bacterial Infection (P354) 4. Meningitis (Serious Infection) (P352) 5. Neonatal tetanus (A33) 6. Pneumonia (Serious Infection) (P353) 7. Preterm delivery (<33 weeks GA) without Respiratory Distress Syndrome (O601)…………….. 8. Respiratory distress syndrome (<33 wks GA) (O602)… 9. Respiratory distress syndrome (33-36 wks GA) (O603) 10. Sepsis (Serious Infection) (P351) 11. Stillbirth (Z37)………………………………………………     **Neonatal Co-morbid Conditions**  1. Preterm Delivery (without RDS) and Birth Asphyxia (O6011)………..……………………………………………….  2. Preterm Delivery (with or without RDS) and Sepsis (O6012)…………………………..……………………….……  3. Preterm Delivery (without RDS) and Sepsis and Birth Asphyxia (O6013) ……………...……………………………..  **Child Infectious Disease**   1. AIDS (B20) 2. AIDS with TB (C30) 3. Diarrhea (A091) ……………………………….................... 4. Dysentery (A092)……………………………….................. 5. Encephalitis (A83) ……… 6. Hemorrhagic fever (A91) 7. Malaria (B54) 8. Measles (B05) 9. Meningitis (A39) 10. Pertussis (A37)…........................................................... 11. Pneumonia (J12) 12. Sepsis (without local bacterial infection) (S30) 13. Sepsis (with local bacterial infection) (R20) 14. Tuberculosis (A15) | □  □  □  □  □  □  □  □  □  □  □  □  □  □  □  □  □  □  □  □  □  □  □  □  □  □  □  □  □ | **Injury**  1. Bite of Venomous Animal (X20)……………………  2. Drowning (W65)……………………………………..  3. Falls (W00)…………………………………………..  4. Fires (X09)……………………………………………  5. Poisonings (T36)…………………………………….  6. Road Traffic (V99)………………………………...…  7. Violent Death (Y05)…………………………………  **Childhood Co-morbid Conditions**  1. Pneumonia and Diarrhea (P3531) ………………...  **Childhood Residual Categories** (also fill in box below)  1. Other Childhood Infectious Diseases (ZZ11)…….  2. Malignant Neoplasms (ZZ12) ..……………………  3. Cardiovascular Diseases (ZZ13) ...……………….  4. Respiratory Diseases (ZZ14)………………………  5. Digestive Diseases (ZZ15)…………………………  6. Other Defined Causes of Child Deaths (ZZ16)…. | □  □  □  □  □  □  □  □  □  □  □  □  □  □ |
| --- | --- | --- | --- |

RESIDUAL CATEGORY DEATHS

| Category (as listed above):  ______________________ | Cause of Death: ____________________________________________________________  ICD – 10 Code: ____________________________________________________________ |
| --- | --- |

**SECTION 5: ADULT DEATHS ONLY**

**FINAL GOLD STANDARD DIAGNOSIS FOR THE ADULT CAUSES**

***Directions:***  *Using information collected above; mark the appropriate diagnosis for the patient. This page will be submitted to the data entry personnel.*

- 1. Study status

| **ADULT CAUSES**  **Maternal**   1. Hemorrhage (O67)……………………………………… 2. Sepsis (S85)……………………………………………. 3. Hypertensive Disorder (H11)…………………………. 4. Obstructed Labor (O64)……………………………….. 5. Anemia (W15)……………………………………………   **Adult Infectious Disease**   1. AIDS (B20)……………………………………………… 2. AIDS with TB (C30)…………………………………….. 3. Diarrhea (A091).............................................................. 4. Dysentery (A092)…….................………………………… 5. Pneumonia (J12)……………………………………….. 6. TB (A15)…………………………………………………. 7. Malaria (B54)……………………………………………. 8. Pelvic Inflammatory Disease (N77)……………………   **Cancers**   1. Breast Cancer (C50)……………………………………. 2. Cervical Cancer (D05)……………………………......... 3. Colorectal Cancer (G18)……………………………….. 4. Liver Cancer (K22)……………………………………… 5. Lung Cancer (C34)……………………………………… 6. Stomach Cancer (A16)…………………………………. 7. Esophageal Cancer (C15)…………………….............. 8. Leukemia (D91)………………………………………… 9. Lymphomas (G96)……………………………………… 10. Mouth/Orapharynx Cancer (C13)…………….............. 11. Ovarian Cancer (F56)………………………………….. 12. Prostate Cancer (H61)…………………………………. 13. Uterine Cancer (C55)……………………………………   **Co-morbid Conditions** (can be checked in addition to other GS cause of death; see Appendix A of the Adult VA Gold Standard list):  Diabetes (E10)  Hypertension (I10) | □  □  □  □  □  □  □  □  □  □  □  □  □  □  □  □  □  □  □  □  □  □  □  □  □ | **Adult Non-Communicable Disease**   1. Cirrhosis (K71)…………………………………... 2. COPD (J33)……………………………………… 3. IHD – Acute Myocardial Infarction (I21)……… 4. IHD – Congestive Heart Failure (T50)………… 5. Renal Failure (N17)……………………………... 6. Stroke (I64)………………………………………. 7. Asthma (J45)…………………………………….. 8. Dementia (F03)………………………………….. 9. Diabetes with Coma (E11)…………………….. 10. Diabetes with Renal Failure (M14)…………… 11. Diabetes with Skin Infection/Sepsis (Q10)….. 12. Epilepsy (G40)………………………………….. 13. Cardiomyopathy (Inflammatory Heart Disease) (I52)…………………………………………........... 14. Endocarditis (Inflammatory Heart Disease) (I52)………………………………………………… 15. Pericarditis (Inflammatory Heart Disease) (I52)…………………………………………………   **Injury**   1. Bite of Venomous Animal (X20)………………… 2. Drowning (W65)…………………………………... 3. Falls (W00)………………………………………… 4. Fires (X09)……………………………………….... 5. Poisonings (T36)………………………………….. 6. Road Traffic (V99)………………………………… 7. Homicide (Y00)………………………………….... 8. Suicide (X70)………………………………….......   **Residual Categories** (also fill in box below)   1. Other Infectious Diseases (ZZ21)...…………….. 2. Other Defined Cancers (ZZ22)...………………... 3. Other Specified Cardiovascular Diseases (ZZ23)………………………………………………. 4. Other Specified Digestive Diseases (ZZ24)...…. 5. Other Non-communicable Diseases (ZZ25)....... 6. Other Defined Causes of Death as a Consequence of Pregnancy (ZZ26)..…………… 7. Other Injuries (ZZ27)...…………………………… | □  □  □  □  □  □  □  □  □  □  □  □  □  □  □  □  □  □  □  □  □  □  □  □  □  □  □  □  □  □ |
| --- | --- | --- | --- |

RESIDUAL CATEGORY DEATHS

| Category (as listed above):  ______________________ | Cause of Death: ____________________________________________________________  ICD – 10 Code: ____________________________________________________________ |
| --- | --- |
